# Quantifying the economic impact of government and charity funding of medical research on private research and development funding in the United Kingdom

**DOI:** 10.1186/s12916-016-0564-z

**Published:** 2016-02-24

**Authors:** Jon Sussex, Yan Feng, Jorge Mestre-Ferrandiz, Michele Pistollato, Marco Hafner, Peter Burridge, Jonathan Grant

**Affiliations:** RAND Europe, Cambridge, UK; Formerly, Office of Health Economics, London, UK; Office of Health Economics, London, UK; Department of Economics, University of York, Heslington, UK; Policy Institute at King’s, King’s College London, Strand Campus, London, WC2R 2LS UK

**Keywords:** Biomedical and health research, Elasticities, Medical research charities, Pharmaceuticals, Rate of return, Research investment, Value of health, Vector Error Correction Model

## Abstract

**Background:**

Government- and charity-funded medical research and private sector research and development (R&D) are widely held to be complements. The only attempts to measure this complementarity so far have used data from the United States of America and are inevitably increasingly out of date. This study estimates the magnitude of the effect of government and charity biomedical and health research expenditure in the United Kingdom (UK), separately and in total, on subsequent private pharmaceutical sector R&D expenditure in the UK.

**Methods:**

The results for this study are obtained by fitting an econometric vector error correction model (VECM) to time series for biomedical and health R&D expenditure in the UK for ten disease areas (including ‘other’) for the government, charity and private sectors. The VECM model describes the relationship between public (i.e. government and charities combined) sector expenditure, private sector expenditure and global pharmaceutical sales as a combination of a long-term equilibrium and short-term movements.

**Results:**

There is a statistically significant complementary relationship between public biomedical and health research expenditure and private pharmaceutical R&D expenditure. A 1 % increase in public sector expenditure is associated in the best-fit model with a 0.81 % increase in private sector expenditure. Sensitivity analysis produces a similar and statistically significant result with a slightly smaller positive elasticity of 0.68. Overall, every additional £1 of public research expenditure is associated with an additional £0.83–£1.07 of private sector R&D spend in the UK; 44 % of that additional private sector expenditure occurs within 1 year, with the remainder accumulating over decades. This spillover effect implies a real annual rate of return (in terms of economic impact) to public biomedical and health research in the UK of 15–18 %. When combined with previous estimates of the health gain that results from public medical research in cancer and cardiovascular disease, the total rate of return would be around 24–28 %.

**Conclusion:**

Overall, this suggests that government and charity funded research in the UK crowds in additional private sector R&D in the UK. The implied historical returns from UK government and charity funded investment in medical research in the UK compare favourably with the rates of return achieved on investments in the rest of the UK economy and are greatly in excess of the 3.5 % real annual rate of return required by the UK government to public investments generally.

**Electronic supplementary material:**

The online version of this article (doi:10.1186/s12916-016-0564-z) contains supplementary material, which is available to authorized users.

## Background

### Estimating the rate of return (RoR) from public investment in biomedical and health research

Realising benefits from medical research in terms of preventing or treating illness, advancing scientific knowledge and generating economic wealth often, although not always, involves private industry [1, 2]. The private sector builds on and interacts with government- and charity-funded research and researchers; it conducts its own further research, and develops and commercialises medicines and other technologies for use in healthcare. Theoretical and applied analyses thus far published predominantly imply that government- and charity-funded medical research and private sector research and development (R&D) are complements [3–7]. In other words, extra spending on medical research stimulates, or ‘crowds in’, extra private sector investment in R&D (and potentially vice versa) and does not substitute for, or ‘crowd out’, private R&D. However, thus far, the only attempts to measure this complementarity have used data from the United States of America (US) and are inevitably increasingly out of date [5, 6].

The purpose of the study reported herein is to estimate the magnitude of the effect of government and charity biomedical and health research expenditure in the United Kingdom (UK), separately and in total, on subsequent private pharmaceutical sector R&D expenditure in the UK.

The importance of developing contemporary estimates for how much government and charitable biomedical and health research investments (referred to in aggregate as ‘public’ research in the rest of this paper) stimulates private sector activity, has been highlighted in two previous UK studies looking at the economic returns from cardiovascular [4] and cancer [8] research, respectively. Both of these studies used a ‘bottom up’ method to estimate the net monetary benefit of health gains arising from historical public research investments. For the cardiovascular disease (CVD) study the RoR from health gains was estimated to be approximately 9 %^1^ and for cancer approximately 10 %. In both cases, the RoR from health gains can be added to an estimate of the broader economic impact on gross domestic product (GDP) of the order of 30 %, to give a total RoR of 39 % and 40 % per annum, respectively, figures that have been widely cited in the policy dialogue justifying government investment in research in the UK [9–12]. However, as acknowledged in both of those studies [4, 8], although the GDP gain accounts for three-quarters of the total return, it is a highly uncertain figure, based on a small amount of empirical literature that is US-centric and not necessarily focused on biomedical and health research.

### A conceptual framework for estimating GDP gains

**Fig. 1:**
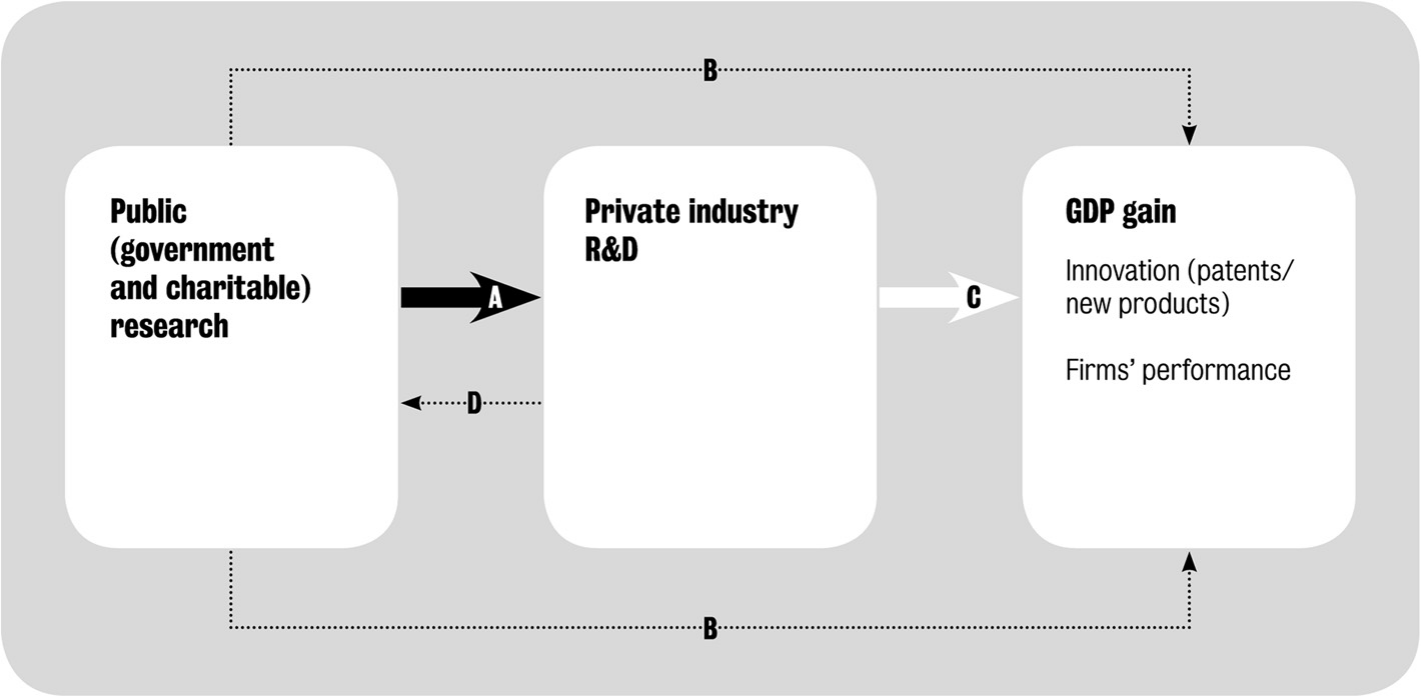
Conceptual model illustrating how public research interacts with private research and development

In the original study on CVD disease [4], two of the authors of this paper (JS, JM-F) developed a conceptual framework (Fig. 1) to estimate the economic gain resulting from government and charity investments in biomedical and health research. These investments can have a direct effect on GDP (represented by the arrows labelled B in Fig. 1) or can be mediated via the private sector. In an economy such as the UK’s, the private sector is likely to be the channel via which the majority of economic impact is mediated; the most recent data show that government services contributed 23 % of UK GDP in 2014 [13] and the charity sector was 0.9 % of UK GDP in 2011/12 [14].

In the current study, we are specifically interested in measuring the arrow labelled A in Fig. 1, which relates to the study objectives to estimate the magnitude of the effect of government and charity biomedical and health research expenditure in the UK, separately and in total, on subsequent private pharmaceutical sector R&D expenditure in the UK. It is possible that an exogenous increase in private R&D could have an impact on public research spending; and we hope to investigate that relationship (arrow D in Fig. 1) in a future study. In the language of economics we are assessing whether public and private investments in medical research are complements to, or substitutes for, one another. That is, we investigate whether, in the UK, public research stimulates additional private R&D spending or replaces it.

The effect of public research spending on private industry R&D may then be multiplied by the social RoR to the additional private sector investment to estimate the total economic impact. This is illustrated as arrow C in Fig. 1. The social RoR includes both the economic benefit to the private firms who are making the R&D investments (the ‘private RoR’) and the spillover effects on the rest of the economy. The term ‘spillover’ is used by economists to describe an investment by one organisation, public or private, that benefits not only that organisation but also other organisations in the same sector of the economy (in this case the life sciences sector) and in any other sector of the economy [15]. In principle, spillovers can also be to other countries, but our study is limited to the impact within one country, the UK. It is important not to view spillovers as accidental, as they are often a deliberate policy objective of spending on public research.

### Existing empirical estimates

There are two key studies, both using US data, that estimate the magnitude of arrow A in Fig. 1. The more recent is by Toole [5], who provides US estimates for the long-term elasticity of private pharmaceutical industry R&D with respect to publicly (National Institutes of Health, NIH)-funded basic and clinical research separately. According to Toole [5], who used NIH data from 1981 to 1996 and private R&D data from 1981 to 1997, a 1 % increase in basic research expenditure by NIH leads to a cumulative 1.69 % increase in pharmaceutical industry R&D spend that builds up over 8 years. For a 1 % increase in NIH-funded clinical research the increase in private R&D is smaller (0.40 %) but occurs more quickly, after 3 years. To translate these estimates to the UK context, the HERG et al. study [4] assumed a 50:50 split in the UK between basic and clinical research funded by the government and charities; hence Toole’s findings imply, on that basis, that in the UK a 1 % increase in public research would produce a (0.5 × 1.69 + 0.5 × 0.40 =) 1.05 % increase in private sector R&D expenditure.

The other key study, by Ward and Dranove [6], did not distinguish between basic and clinical research but also focused on US NIH funding. This older study, using US data from 1966 to 1988, implies a considerably larger impact of US public medical research on private pharmaceutical industry R&D than was found by Toole [5]. Ward and Dranove [6] estimated that a 1 % increase in NIH spend across all therapeutic areas leads to a 2.50 % increase in the total of private pharmaceutical R&D spend in the US that builds up over 7 years.

This additional private R&D spend stimulated by public research expenditure has both private benefits (i.e. to the company making the investment) and other social benefits (i.e. to wider society) as captured by arrow C in Fig. 1. In the HERG et al. study [4], 11 empirical papers were identified that used various methods to estimate the social RoR on private R&D. From this evidence, the social RoR to private sector R&D expenditure was estimated to be around 50 % [4]. Combining that social RoR figure to private R&D with, respectively, the Toole [5] and Ward and Dranove [6] estimated elasticities of private R&D to public medical research spend (and of the time lags involved), implied a real annual economic RoR to the original public investment of 26–34 %, which was presented in HERG et al. as “around 30 %” [4].

Since the HERG et al. study [4], two relevant reports have been published. The first by Frontier Economics [16], reviewed the literature on RoR to investment on science and innovation. A lot of this literature was covered by HERG et al. [4], with the Frontier Economics study [16] effectively updating this review. It concluded that the social returns to publicly-funded R&D investments have found significant, positive returns of around 30–40 %, but this was largely based on the agricultural sector and international evidence. Frontier Economics cited a recent study by Haskel et al. [17], which focused on the UK and looked at how different industrial sectors interact with publicly funded R&D. By modelling the impact of public R&D on private sector productivity, this study estimated positive and significant social returns of around 20 % for UK public R&D investments, but it does not have a specific focus on biomedical and health research. Nevertheless, these two studies are broadly in line with the previous estimate for the RoR in terms of GDP of 30 %, and highlight the absence of UK relevant estimates for biomedical and health research.

### Paper structure

The remainder of this paper describes how we estimated whether and how far public research investments crowd in or crowd out private sector R&D investments for the biomedical and health sciences for the UK (i.e. arrow A in Fig. 1). The next section sets out our methodological approach, including introducing the econometric model that was developed, and details on how we collated and estimated the time series data for the modelling. Our key findings are presented in the results section, including various tests on the strength of the model and a sensitivity analysis. The final section reviews the limitations to our analysis and its broader interpretation in a policy context. We have provided eight Additional Files that provide access to the underlying data, and various steps in deriving them, as well as a literature review of drivers of public and private R&D.

## Methods

### Estimating the relationship between public and private R&D expenditures in the UK

The results for this study are obtained by fitting an econometric vector error correction model (VECM) [18] to time series for biomedical and health R&D expenditure in the UK for ten disease areas (including ‘other’) for the government, charity and private sectors. It should be noted at the outset, and as discussed further in Box 1, that an econometric model specifies the statistical relationship that exists between various time series and is only as good as the data that are used and the abstraction of the underlying phenomenon under study. For an accessible introduction to empirical model building in economics see Granger [19]. The VECM used describes the relationship between public (i.e. government and charities combined) sector expenditure, private sector expenditure and global pharmaceutical sales as a combination of a long-term equilibrium and short-term movements. As noted below, we undertook a brief policy and literature review partly to ensure that we were aware of, and if appropriate, took into account any major ‘shocks’ from the external policy environment that could impact on our model. As a result, we included global pharmaceutical sales in this model of long-term equilibrium because our literature review identified ‘potential market size’ as a key driver of private R&D, which we proxied by global pharmaceutical sales (by therapy area).

### Targeted policy and literature reviews

In the early stages of the project, we undertook three targeted reviews of the policy and academic literature. The first was to update the literature review undertaken for the HERG et al. study [4], and this identified two additional papers as cited earlier [16, 17]. The second was to see whether we could identify any significant regulatory events that might have had an impact on R&D (private, public, charitable) investment decisions. We anticipated that, as part of the modelling exercise, we might need to test for ‘dummy’ or categorical variables where there was a major change in policy that could affect the results of the econometric modelling. The third was for drivers for public and private R&D funding as these might need to be considered as control variables in the econometric modelling. All reviews were undertaken by using a range of search terms in various search engines and bibliographic databases and ‘snowballing’ from that seed literature as necessary. We should stress that this was not intended to be a systematic review, but was designed to inform model developments.

### Developing R&D investment time series

To measure the relationship between government, charitable and privately funded R&D we built a 31-year set of time series comprising UK data for government, charitable and private sector R&D spend from 1982 to 2012 for nine different disease areas plus ‘other’ (the sum of all other disease areas apart from the nine). We needed multiple time series (i.e. by source of funding and disease area) to ensure enough statistical variation in the modelling, but not so many as to create an unduly large task of trying to identify public (and especially charity) research expenditures for each of them. The selected definitions were a consequence of the categories for which charitable and government expenditure data could be distinguished, while being sufficiently aggregated to minimise the risk of significant changes in the definitions of disease area specific data over time. As illustrated in Table 1, we focused our analysis on: Blood; Cancer; Cardiovascular; Central Nervous System (CNS); Gastroenterology; Infection; Respiratory; Skin; Vision; and Other. These areas were selected as there was a relatively strong ‘mapping’ between a historic classification system used by the Medical Research Council (MRC), the more contemporary Health Research Classification System (HRCS) definitions (used by funders in the UK) [20], and Thomson Reuters Journal Subject Classifications [21], used in bibliometric analysis as discussed below. Moreover, some of the areas were small (e.g. Vision) and others large (e.g. Cancer) adding to the variability in the data, which enhances the modelling. In estimating these 10 time series we used a similar approach to that developed by HERG et al. [4] and Glover et al. [8] for CVD and Cancer, respectively. Throughout our analysis we used the UK GDP deflator at market prices to convert all monetary values to constant 2012 prices [22].

**Table 1:** Definitions of field by different classifications

Field	Medical Research Council	HRCS ^a^	Thomson Reuters JSC ^b^
Blood	Blood: red cells (erythrocytes); white cells and reticuloendothelial system (including bone-marrow); platelets and coagulation (thrombosis); serum proteins (antibody, etc.); and inflammatory systems (allergy, histamine, oxytocin, vasoactive agents)	Blood: Diseases caused by pathogens, acquired immune deficiency syndrome, sexually transmitted infections, and studies of infection and infectious agents	Haematology covers resources that deal with blood and blood-forming tissues, as well as the functions, diseases, and treatments of these systems. Topics included are haemophilia, neoplastic disorders of the blood or lymphoid tissues, and mechanisms and disorders of thrombosis
Cancer	Cancer: Carcinogenesis (chemical and physical substances, ionising radiation, asbestos, mutagens, occupational medicine); incidence/epidemiology; detection/diagnosis, tumour biology, radiotherapy (radiobiology, adjuvants); chemotherapy (drugs, therapeutics techniques – side effects); and immunotherapy (immunotherapy)	Cancer: All types of cancers (includes leukaemia)	Oncology covers resources on the mechanisms, causes, and treatments of cancer including environmental and genetic risk factors, and cellular and molecular carcinogenesis. Aspects of clinical oncology covered include surgical, radiological, chemical, and palliative care; this category is also concerned with resources on cancers of specific systems and organs
Cardiovascular	Cardiovascular: heart (electrophysiology); veins (vasoactive agents); arteries (cerebrovascular, arteriosclerosis, vasoactive agents); lymphatics (white cells); hormonal and metabolic systems (metabolism, electrolytes, hormones, oxytocin, steroids, vasoactive agents)	Cardiovascular ^c^: Coronary heart disease, diseases of the vasculature and circulation system including the lymphatic system, and normal development and function of the cardiovascular system	Cardiac and Cardiovascular Systems covers resources dealing with the diagnosis and treatment of heart disease; coverage focuses on cardiac disease prevention, pharmacology, surgery, transplantation, and research. This category also includes cardiac testing, pacemakers, and medical devices. Resources focusing on circulation, hypertension, arterial disease, and stroke are placed in the peripheral vascular disease category
Central Nervous System	Central Nervous System: Mental health and mental disorders; electro-physiology; epilepsy, head, Huntington’s chorea, migraine, multiple sclerosis, rabies, and transmitters	Neurological ^d^: Dementias, transmissible spongiform encephalopathies, Parkinson’s disease, neurodegenerative diseases, Alzheimer’s disease, epilepsy, multiple sclerosis and studies of the normal brain and nervous system	Neurosciences covers resources on all areas of basic research on the brain, neural physiology, and function in health and disease. The areas of focus include neurotransmitters, neuropeptides, neurochemistry, neural development, and neural behaviour. Coverage also includes resources in neuro-endocrine and neuro-immune systems, somatosensory system, motor system and sensory motor integration, autonomic system as well as diseases of the nervous system
Gastroenterology	Gastrointestinal: Mouth and pharynx (salivary gland, tonsils and adenoids); oesophagus and stomach (foodstuffs (hazards and constituents)); small intestine (coeliac disease); colon and rectum (incontinence); hepatobiliary system (metabolism – lipids, hepatitis), and exocrine pancreas (cystic fibrosis)	Oral and Gastrointestinal: Inflammatory bowel disease, Crohn’s disease, diseases of the mouth, teeth, oesophagus, digestive system including liver and colon, and normal oral and gastrointestinal development and function	Gastroenterology and Hepatology covers resources on the anatomy, physiology, biochemistry, and pathology of the digestive system. This category includes specific resources on the prognosis and treatment of digestive diseases, stomach ulcers, metabolic, genetic, infectious and chemically induced diseases of the liver, colitis, diseases of the pancreas and diseases of the rectum
Infection	Infections: Viral and mycoplasmal (phage and virus, common cold, cross-infection, hepatitis, herpes, influenza, interferon, measles, poliomyelitis, rabies, rubella); bacterial and rickettsial (bacterial cells, antibiotics, cross-infection, drug resistance, venereal diseases, whooping cough); mycobacterial, fungal leprosy, tropical and overseas, tuberculosis); yeast, protozoal (malaria, tropical and overseas, vectors); Helminth diseases (molluscs, tropical and overseas, vectors)	Infection: Diseases caused by pathogens, acquired immune deficiency syndrome, sexually transmitted infections, and studies of infection and infectious agents	Infectious Diseases covers resources on all aspects of the pathogenesis of clinically significant viral or bacterial diseases including HIV, AIDS, sexually transmitted diseases; this category is also concerned with resources on host-pathogen interactions, as well as the prevention, diagnosis, treatment, and epidemiology of infectious disease
Respiratory	Respiratory: Upper respiratory tract (including epiglottis and larynx) (common cold, influenza); airways and lungs (allergy, asbestos, asthma, bronchitis, pneumoconiosis, tuberculosis, whooping cough)	Respiratory: Asthma, chronic obstructive pulmonary disease, respiratory diseases, and normal development and function of the respiratory system	Respiratory System covers resources on all aspects of respiratory and lung diseases, including their relation to cardiovascular and thoracic surgery and diseases
Skin	Skin: Allergy, leprosy, psoriasis, and venereal diseases	Skin: Dermatological conditions and normal skin development and function	Dermatology covers resources on the anatomy, physiology, and pathology of the skin. It contains resources on investigative and experimental dermatology, contact dermatitis, dermatologic surgery, dermatologic pathology, and dermatologic oncology; tis category also includes specific resources on burns, wounds and leprosy
Vision	Vision: Electrophysiology, eye, retinitis pigmentosa	Eye: Diseases of the eye and normal eye development and function	Ophthalmology covers resources on the eye, its diseases, and refractive errors; coverage includes research on the cornea, retina, and eye diseases. This category also includes resources on physiological optics and optometry as well as reconstructive surgery

^a^ Health Research Classification System (http://www.hrcsonline.net/hc/view)

^b^ Thomson Reuters Journal Subject Classifications http://ip-science.thomsonreuters.com/mjl/scope/scope_scie/

^c^ There is a Stroke classification: Ischaemic and haemorrhagic

^d^ There is a Mental Health classification: Depression, schizophrenia, psychosis and personality disorders, addiction, suicide, anxiety, eating disorders, learning disabilities, autistic spectrum disorders and studies of normal psychology, cognitive function and behaviour

### Government expenditure on biomedical and health R&D in the UK, 1982–2012

In the UK, the main government investors in biomedical and health R&D are the MRC, the Department of Health (DH) and the Funding Councils. Below we explain how we estimated R&D expenditure for these three research funders between 1982 and 2012.^2^

#### Medical Research Council (MRC)

Since 1970, the MRC used different systems for classifying grants. For our analysis, we use the longest time series for which a comparable classification is available, which is between 1976/77 and 1992/93 and then subsequently from 2006 and 2012. For this period, annual spend on funded grants was classified in two ways. The first was based on the primary purpose of the research and provided an ‘exclusive’ measure of spend by a number of headings. The second was a more ‘inclusive’ measure where spend could be placed against a number of different categories. Using this classification system, a ‘Breakdown of MRC expenditure by subject heading’ is annexed in the MRC’s annual reports between 1976/77 to 1992/93. In this study, we used the ‘exclusive’ measure, as we wanted to avoid double counting between research areas^3^ and the subject headings as detailed in Table 1. For the period after 2006, the MRC uses the HRCS. For the intervening period between 1993/94 and 2006 the data are interpolated using an exponential function.

#### Department of Health (DH)

The DH data include the National Health Service (NHS; only collected in official statistics from 1995 onwards) and DH expenditure on the policy research programme for the UK (this includes National Institute of Health Research expenditure from 2006/07). Using 1995/96 to 2011/12 NHS data published in the online version of the Science, Engineering and Technology (SET) Statistics [23], we extrapolated backwards the series for the years 1982–1994 and extrapolated the value for 2012/13 using an exponential function. What is more, we generated a DH time series by supplementing the DH data for 1986/87 to 2011/12 published in the online version of the SET Statistics with information for 1982/83 to 1985/86 from a published report of the SET Statistics [24]. As for the NHS data we extrapolated the 2012/13 values using an exponential function. We then added the NHS and DH data series together.

#### Funding councils

The Higher Education Funding Council for England (HEFCE) directly provided us with funding data for biomedical subjects from 1989/90 to 2012/13. The data for 1989/90 to 1992/93 were for the predecessor body, the University Funding Council, and covered Great Britain. From 1993/94 onwards the data are available for England alone. Biomedical research was defined by the cost centres/units of assessment used at the time. In order to estimate a time series for 1982–2012, we used HEFCE data for the period 1989–2012 (with adjustments to include data for Wales, Scotland and Northern Ireland where otherwise not taken into account) and then extrapolated these figures backwards using an exponential function to generate data for the period in question.

The DH and Funding Council data are not available by disease area. We therefore used bibliometric data on publications in the nine disease areas provided by the Centre for Science and Technology Studies (CWTS)^4^ at Leiden University (Additional file 1). In essence, we multiplied the total spend of the DH and the total biomedical spend of the Funding Councils by the proportion of peer review research papers with no private industry author by disease area to proxy estimated DH and Funding Council research spend by disease area.

### Charitable expenditure on biomedical and health R&D in the UK, 1982–2012

We draw expenditure data on biomedical research for the charitable sector from two sources: the Wellcome Trust and the Association of Medical Research Charities (AMRC) (excluding the Wellcome Trust). To be in line with government expenditure data, we use ‘active in’ (in contrast to ‘awarded in’ figures) for research grants provided by the charitable sector sources.

#### Wellcome Trust

Wellcome Trust expenditure on research is derived from the Wellcome Trust grants database using a combination of keyword searches and classification terms developed and used by the Trust. Historically, grants have been classified by Grant Officers using a thesaurus of terms. A list of search terms was developed for the nine disease areas of investigation applied to the titles of awarded grants (Additional file 2). The time series for ‘Cancer’ was available since 1981 due to earlier work [8], whereas the series for all the other disease areas start at some point in the early 1990s. All the series, except for Cancer, have been backward extrapolated between 1982 and the early 1990s using an exponential function.

#### Association of Medical Research Charities (AMRC)

The AMRC directly provided us with funding data for all UK medical research charities except for the Wellcome Trust, for which we already had direct data available. An AMRC data scientist coded the charities according to their main activity into one of the nine disease areas according to the HRCS classification (see Additional file 3 for details). Note that data on funding streams is generally available from 1992 onwards and we backward extrapolated the values from 1982 to 1992 using an exponential function. Note that no charities with main activity for the disease area ‘Blood’ were detected in the AMRC database.

### Private expenditure on biomedical and health R&D in the UK, 1982–2012

The Association of the British Pharmaceutical Industry compiles and publishes data on total UK R&D expenditure by the pharmaceutical industry. Unfortunately, a disaggregation by particular disease area is not directly available. Similar to the approach to generate a time series by disease area for the DH and Funding Councils data, we use a proxy for research activity. We multiply the total UK pharmaceutical industry R&D spend by year with the proportion of publications with authors giving UK industry addresses by disease area. To that end, we use an assumed 4-year lag between R&D expenditure and consequent publication – we elaborate on the reason for that particular assumption in the econometric modelling section (see section ‘Best model’ for more details and Additional file 1 for the bibliometric data).

#### Global pharmaceutical sales

As will be discussed in the results section, the literature review (see Additional File 4 for more details) identified global market size as a key driver of private pharmaceutical R&D [25–28]. The published literature has modelled in various ways the variable ‘market size’; for our purposes, we use as a proxy ‘global pharmaceutical sales’ per disease area. The source for such data is IMS Health, a global information and technology services company in the healthcare industry. In particular, we have used IMS Health’s Annual World Reviews to extract global pharmaceutical sales, by disease area, from 1977 to 2012 [29].

We did this in stages, to match our nine disease areas (Table 1, as above). IMS Health provides sales data at Anatomical Therapeutic Classification (ATC) level, so the first stage was to match the ATC codes with our disease areas. In the ATC classification system, the active substances are divided into different groups according to the organ or system on which they act and their therapeutic, pharmacological and chemical properties. Drugs are classified in groups at five different levels. The drugs are divided into 14 main groups (first level), with pharmacological/therapeutic subgroups (second level). The third and fourth levels are chemical/pharmacological/therapeutic subgroups and the fifth level is the chemical substance. More information on ATC codes can be found at: http://www.whocc.no/atc/structure_and_principles/. The nine therapy areas and codes are provided in Table 2 (noting that we had to include a selection of ATC2 codes for some ATC1 classes).

**Table 2:** Matching our nine disease areas with Anatomical Therapeutic Classification (ATC) codes

Therapy area	ATC 1 and ATC2 level
Gastroenterology	A
Blood	B
Cardiovascular	C
Skin	D
Cancer	L01; L02^a^
Central Nervous System	N03; N04; N05; N06; N07^b^
Infection	J, P^c^
Respiratory	R
Vision	S01^d^

^a^ L contains four ATC2 codes, but cancer drugs are only included in L01 (Antineoplastic agents) and L02 (Endocrine therapy). L03 and L04 refer to Immunostimulants and Immunosuppresants, respectively

^b^ N contains seven ATC2 codes, but we have only included the following five ATC2 codes: N03 Antiepileptics; N04 Anti-Parkinson drugs; N05 Psycholeptics; N06 Psychoanaleptics; N07 Other nervous system drugs. N01 refer to Anesthetics and N02 to Analgesics

^c^ *J* Anti-infectives for systemic use, *P* Antiparasitic products, insecticides and repellents

^d^ Vision does not have a separate ATC1 code, and thus we used an ATC2 code (S01, Ophthalmologicals)

We had access to IMS Annual World Reviews since 1977. In each Review, the top 100 therapeutic areas by sales (global), at ATC3 level, are included. For every year between 1977 and 2012, we matched our ATC1 and ATC2 codes in Table 2 with the ATC3 codes included in the top 100 list, and summed across ATC3 codes where appropriate, to obtain global sales per year for each of the nine disease areas above. The Reviews also provide a figure for the total global pharmaceutical market; we thus obtained global sales for the ‘Other’ category by subtracting the sum of sales across the nine therapy areas from the global figure.

It should be noted that, over the years, the coverage by IMS Health of the global market has increased. We are not aware of all the nuances around increased coverage, but it would have expanded in terms of coverage within any one country (for instance, including hospital sales rather than sales at pharmacy level), and across countries (including more countries over time). However, we expect that the increased coverage over time would not bias our results, as we are primarily interested in the relative shares of global sales across our nine therapy areas.

### Key informant interviews

We undertook a small number of key informant interviews to validate and test our use of bibliometric indicators of research activity to proxy the therapy area split of pharmaceutical industry R&D expenditure in the UK, and to ask for interviewees’ views on the drivers of their company’s R&D and its interrelationship with government- and charity-funded medical research. We contacted senior research managers from five pharmaceutical companies with major research facilities in the UK and/or funding substantial research activity (clinical trials, etc.) in the UK. Four responded and were interviewed. The interview protocol is available in Additional file 5.

### VECM specification

All data analyses and econometric modelling were conducted in EViews 8.0 [30].

#### Variables

To construct the VECM (for a quick introduction to the technical issues see Enders [31] and Garratt et al. [32]), we first run Augmented Dickey Fuller (ADF) unit root tests to check the non-stationarity of the five time series [33], i.e. government biomedical and health research expenditure, charity biomedical and health research expenditure, public biomedical and health expenditure (the sum of the government and charity expenditures), private sector pharmaceutical R&D expenditure, and global pharmaceutical sales. The ADF test is applied to the levels and first differences, with and without taking logarithms, of all variables. In the finally chosen VECM we use variables in log form, which allows estimated coefficients to be interpreted as elasticities. In the best-fit model, government expenditure and charity sector expenditure are combined into a single measure of public expenditure. We also adapt the best model into a specification that treats government expenditure and charity sector expenditure separately as two variables.

#### Model

The VECM treats all variables as endogenous. Whether the model is appropriate for our data is an empirical question. The model details are chosen by a specification search. First, the number (0, 1 or 2) of cointegration relationships (long-term equilibrium relations) between public sector research expenditure, private sector R&D expenditure and global pharmaceutical sales is determined. Were this to be 0, other models would have to be considered. Second, the number of time lags needed to properly account for the short-term movements of each of the three variables is determined. Third, the presence or absence of a deterministic trend for both the long-term and short-term effects must be decided. Whether there are cointegration relationships between the three variables is the key issue for the specification of a VECM. Since the cointegration test outcomes are in general affected by the number of lags included for the short-term effect and specification of the deterministic trend, the three model features must be determined simultaneously.

#### Model search

Mindful of the moderate time span of our data (31 years), the model was estimated with one, two or three lags in the short-run dynamics [34–36]. For each case, four specifications of the deterministic trend were tested [37]. The first assumes that there is no intercept or trend in the cointegration equation (CE) and the vector autoregression (VAR). The second assumes that there is no trend in the CE or the VAR, but there is an intercept in the CE. The third assumes that there is an intercept in the CE and both an intercept and a trend in the VAR. The fourth specification assumes that there is a trend and intercept in the CE, and an intercept but no trend in the VAR. Results of cointegration tests from each of the 12 specifications show whether there are 0, 1 or 2 cointegration relationships between the three variables. The Pantula principle is then applied to select the preferred model. The selection accounts for a number of factors, i.e. the number of cointegration relationships, autocorrelation of the VECM residuals, number of insignificant coefficients in the VECM and statistics that measure the relative quality of models (Akaike Information Criterion, Schwarz Criterion and log likelihood ratios) [38].

#### Best model

As already noted, for private sector expenditure and for some public expenditure we have data at aggregate level but not disease level. We use the proportion of publications in each disease area as a proxy to calculate the disease level expenditures from the private and public sectors (Additional file 1). It is suggested in the literature that 3 years is the median time elapsed from the start of a new US public medical research grant and the date of the first publication [39]. We tested models with time lags from R&D expenditure to publication assumed to be, respectively, from 0 to 5 years, and selected the lag that gave the best fit. In this exercise, we combine charity expenditure and government expenditure together as one variable, which we call public expenditure. In a subsequent step, the best-fit model is expanded to a specification that treats the two variables as separate, i.e. charity expenditure and government expenditure separately.

#### Sensitivity analysis

As reported below, we reviewed the policy literature and considered the 1984 US Hatch-Waxman Act as the only single event likely to be big enough to have had a perceptible effect [40]. However, we did not include a policy dummy 1984 in our econometric analysis owing to the paucity of pre-1984 data. We noticed that a particularly large increase in charity and government research expenditure occurred in 1993 and so decided to test the effect of including two policy dummies for 1993 as exogenous variables into our model as a sensitivity analysis. The first dummy takes a value of zero if an observation refers to a year before 1993, otherwise it is recorded as one. The second dummy takes a value of one if an observation refers to the year 1993, otherwise it is recorded as zero. Thus, the first dummy would identify any persistent effect and the second dummy any pulse effect.

#### Prediction by the impulse response function

The effect of a permanent shock to public research expenditure on the level of private R&D expenditure is exhibited by the Impulse Response Function [41, 42]. The result shows how private expenditure responds to a maintained shock to public expenditure in both the short and long run. It also shows how private sector expenditure moves back to equilibrium after a maintained shock to public sector expenditure.

## Results

### The relationship between public and private R&D expenditures in the UK

#### Observations arising from the literature review and key informant interviews

As already noted above, there were two key lessons from the policy and literature review that were relevant to the model development (a summary of the literature review is in Additional file 4). First was the role of global pharmaceutical sales as a driver for private sector R&D investment – that is, the higher the level of sales the greater the R&D budget. For this reason we included global pharmaceutical sales as a control variable in the model. Second was the need to allow for a possible ‘dummy’ variable for 1993 due to a large increase in charity and government research expenditure.

The industry-based interviewees supported the use of bibliometrics as the least bad proxy for the therapy area split of their R&D expenditures, but highlighted the approximate nature of that relationship and the time lags involved between R&D spending and consequent publication. They were not able to identify any alternative data on R&D spending by therapy area, either from within their own company or externally.

### Government, charity and private biomedical and health R&D expenditure in the UK, 1982-2012

#### Total UK R&D expenditures (public and private)

**Fig. 2:**
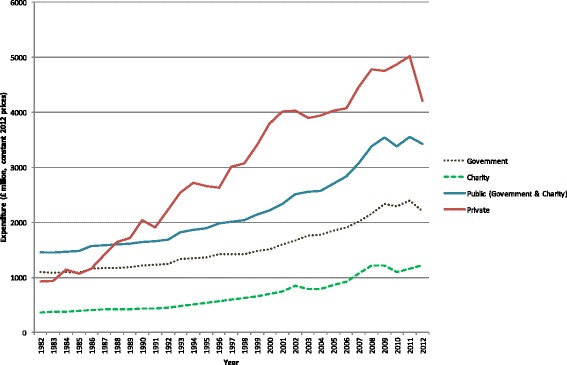
Total UK research and development expenditure (government, charity and private), 1982–2012 (£m, 2012 constant prices)

Figure 2 (accompanied by Additional file 6) reports the total UK biomedical and health research expenditure by the public (government and charity) and the private sector between 1982 and 2012 in constant 2012 prices. We observe a gradual upward trend for the public research expenditure figures. In 1982, UK public R&D expenditure was £1.453bn (in 2012 price terms) and rose to £3.429bn in 2012, which corresponds to a 2.4-fold increase. Disaggregating the public expenditure series into government and charity, UK government sector expenditure rose from £1.093bn to £2.208bn, whereas UK charity sector expenditure increased from £360m to £1.222bn. Between 2002 and 2004, the rise in the public expenditure series comes to a temporary halt, driven by a slower increase in government spending and a decline in the expenditure of the charity sector (from £839m in 2002 to £783m in 2004). Total public expenditure bounces back afterwards and increases steadily after 2004.

Compared to the public research expenditure figures, the data series for private pharmaceutical industry R&D expenditure depicted in Fig. 2 (accompanied by Additional file 6) shows a somewhat steeper upward trend. In 1982, private R&D expenditure was £925m, rising to £4.207bn in 2012, which corresponds to a 4.5-fold increase over the observation period. Furthermore, the figures suggest that private R&D expenditure is subject to more variation than public research expenditure.

#### UK R&D expenditures by disease areas (public and private)

**Fig. 3:**
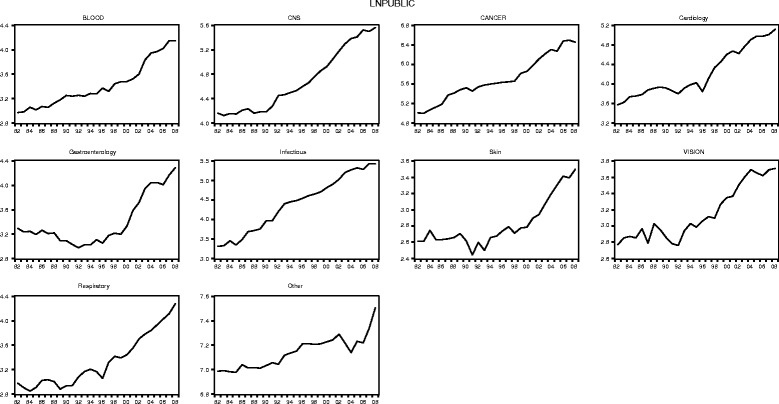
Public (government and charity) research and development (log) expenditure by disease area, 1982–2008 (£m, 2012 constant prices)

**Fig. 4:**
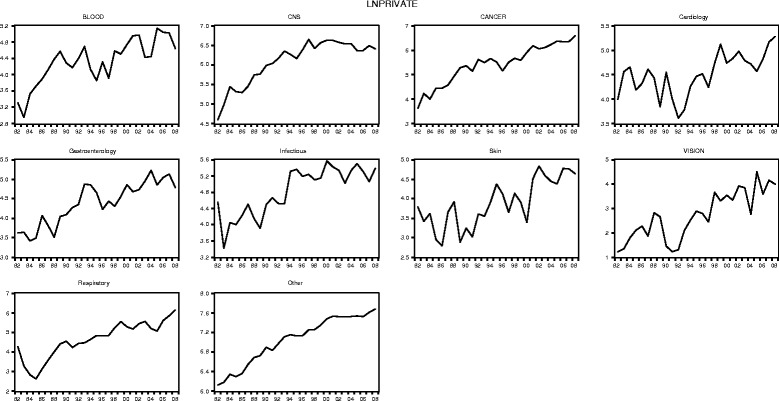
Private research and development (log) expenditure by disease area, 1982–2008 (£m, 2012 constant prices)

Figures 3 and 4 illustrate the public (government and charity) and private R&D expenditure figures by disease area between 1982 and 2008 in the logarithmic form in which they feed into the econometric models. Note that the underlying data is available in Additional file 6 (including public expenditure figures broken down by government and charity).

Similar to the aggregated data series reported in the previous section, at the disease area level we observe, in each case, an overall upward trend in public research and private R&D spending and with more variation, expressed as upward and downward movements in the time series, in the expenditure data for the private sector than for the public sector.

Looking at specific disease areas in more depth, we further observe, for instance, that for ‘Blood’ public R&D expenditure is for almost all years over the observation period smaller than private expenditure (except in year 1983, where there is a fall in private expenditure, followed by a strong upward trend). In ‘CNS’ private expenditure there is an upward trend since 1982 (with short interruptions in 1984 and 1993), which flattens and somewhat reverses with the start of year 1997. In contrast, the trend for public ‘CNS’ expenditure is somewhat flat between 1982 and 1990 and subsequently rises steeply until around 2006. For all years, private R&D expenditure exceeds public R&D in the disease area ‘CNS’. The disease area ‘Cancer’ represents one of the largest areas of biomedical research funding, both for the public and the private sector, and for the majority of years over the observation period, public expenditure related to cancer research exceed private expenditure. The public cancer research series further follows a relatively steady upward trend since 1982, with two smaller interruptions in 1990 and 2004. The private expenditure series for ‘Cancer’ shows more variation over time with two larger interruptions in 1990 and 1995. In ‘Cardiovascular’, the private expenditure series follows no clear trend up until 1996, followed by a small upward trend thereafter but with rather strong variation between 1982 and 2008, with the lowest level of private funding in the area realised in 1992. In contrast, the ‘Cardiovascular’ public R&D expenditure series follows a slightly increasing trend between 1982, interrupted in 1996 and followed by a steady rise until 2008. For the majority of the years in our observation period, private expenditure exceeds public expenditure in cardiovascular research. ‘Gastroenterology’ is an interesting area insofar as public expenditure in the field follows a downward trend until about 1992, increases somewhat thereafter and increasing strongly since 1999. Furthermore, the private expenditure series for ‘Gastroenterology’ shows an upward trend overall with some variation over time (for instance, 1986 and between 1993 and 1996). Similar to the ‘CNS’ expenditure series, private expenditure on gastroenterology research exceeds public expenditure for all years. For the disease area ‘Infectious Diseases’, the public and private R&D expenditure series differ with regard to the trend pattern. Whereas the public expenditure series shows a steady increasing trend since 1982 (with small interruptions in 1984, 1990 and 2005), the private expenditure series shows a lot more variation and a fairly flat trend after 1994. However, for the majority of the years, private R&D still exceeds public research expenditure in the ‘Infectious Diseases’ area. The two smallest disease areas we look at in more depth are ‘Skin’ and ‘Vision’, which both follow similar trends over time. Public expenditure in the two areas is relatively flat until about 1993 and subsequently takes off. In both areas, public expenditure levels are very similar over the observation period. This is different in the private expenditure series, where private spending in ‘Skin’ generally exceeds funding in ‘Vision’, but both private series follow a somewhat upward trend with interruptions. Although private expenditure exceeds public expenditure for all years in ‘Skin’, in ‘Vision’, for the majority of years, public spend exceeds private spend. In the disease area ‘Respiratory’ we observe a relative flat trend in public research, with variation, until around 1997, when a general upward trend in public expenditure in the area kicks in. For the private expenditure series in the respiratory area we observe a relatively strong downturn from 1982 to 1985, followed by an upward trend thereafter. What is more, for the majority of the years, in ‘Respiratory’ private expenditure exceeds public expenditure (except 1984 and 1985).

#### Global pharmaceutical sales

**Fig. 5:**
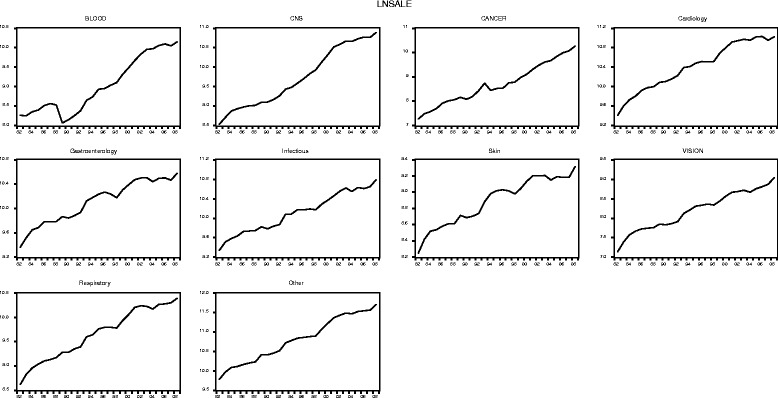
Global pharmaceutical (log) sales by disease area 1982–2008 (£m, 2012 constant prices)

Figure 5 illustrates global pharmaceutical sales (in £m, in 2012 price terms) in logarithmic form by disease area from 1982. Overall, the expenditure patterns in the figure reveal an upward trend in global medicine sales in all the disease areas. However, looking at specific disease areas we observe some variation. For instance in ‘Blood’, there is a decrease in sales from 1988 to 1989, followed by a steady upward trend thereafter. The ‘Cancer’ medicines global sales series somewhat interrupts in 1993, with a decrease in sales between 1993 and 1994, but is followed by a steady increase thereafter. Interestingly, the global pharmaceutical sales series shows a particularly strong rise in sales starting in 1999 in most disease areas.

### Econometric modelling

Overall, our results suggest that there is a statistically significant complementary relationship between public biomedical and health research expenditure and private pharmaceutical R&D expenditure. A 1 % increase in public sector expenditure is associated in the best-fit model with a 0.81 % increase in private sector expenditure. The sensitivity analysis, with dummy variables for 1993 and subsequently, produces a similar and statistically significant result but with a slightly smaller positive elasticity of 0.68.

#### Variables

The ADF unit root test results are reported in Table A in Additional file 7. The null hypothesis for the ADF unit root test is that the individual series is a unit root process. The results suggest that the five variables are non-stationary in levels or logs, but stationary in first differences of levels or logs; we treat the contrary result for log(private) as an artefact of the pooling of individual series outcomes and discount it.

#### Determining the best-fit model

Results from the 12 tested models are reported in Tables B–D in Additional file 7. Each table reports performance of four specifications, one for each deterministic trend specification. In this preliminary search the time-lag between funding and publication is treated as zero.

For each specification of the model we report six statistics: the cointegration rank, the statistics from the autocorrelation test, Akaike Information Criterion, Schwarz criterion, log likelihood statistics, and the number of insignificant coefficients in the VECM. We compare the performance of models using those statistics. The cointegration rank is the estimated number of cointegration relationships (long-term equilibria) between the three variables. ‘Not available’ in Tables B–D in Additional file 7 suggests that there is no cointegration relationship for a specification. Statistics from residual portmanteau autocorrelation tests report the number of lags, with a maximum of six, with absence of a serial correlation problem. The maximum lag 6 tested was chosen pragmatically as the sample is quite short (31 observations for each disease area) and from previous literature [5, 6]. If the number reported is less than 6, a specification problem (serial correlation) is suggested. For the Akaike Information Criterion and Schwarz criterion, the chosen model should be the one that minimises the adopted criterion. Log likelihood statistics parallel the use of information criterion. A larger log likelihood statistic suggests a better model fit. The number of insignificant coefficients shows the number of redundant coefficients in the error correction models in relation to the total number of coefficients in the VECM. We define insignificant coefficients as those with absolute t-values less than 2. The larger the number of insignificant coefficients, the less desirable is the model.

The Pantula principle of parsimony was used to select the best performing model. It picks the most parsimonious model that is not rejected by the cointegration rank tests. The VECM with deterministic trend type 3 and one lag interval performs the best, as shown in the fourth column of Table B in Additional file 7.

Having identified a preferred model, we report results from five variants that allow for different time lags between the date of investment from private sector (and government) and the date of first publication, in Table E of Additional file 7. The basic model is labelled as the t + 0 model. Again, we applied the Pantula principle to select the best model. Only the t + 0 and t + 4 models pass the residual autocorrelation tests for up to six lags. The literature suggests a median of 3 year lag between R&D investment and first publication [39]. Our t + 4 model performed better than t + 3 and t + 5, so we chose the t + 4 model as our best model.

#### Results from the best-fit model

The results presented in Table 3 and Eq. 1 suggest that, in the long run, there is a statistically significant complementary (‘crowding in’) relationship between public biomedical and health expenditure and private pharmaceutical R&D expenditure. The elasticity of private sector expenditure with respect to the public expenditure from each model is reported by the second column of Table 3. This takes the value 0.81 in the best-fit (t + 4) model shown in Table 3, and ranges from 0.38 to 1.12 in the other models.

1 $$ \begin{array}{l}\varDelta {y}_{it}=\Gamma \varDelta {y}_{it-1}+\alpha \left(\beta {y}_{it-1}+\mu \right)+\gamma +{\varepsilon}_{it}\\ {} where\ {y}_{it} = \left[ privat{e}_{it},\  publi{c}_{it},\  sal{e}_{it}\right]'\end{array} $$

**Table 3:** The best model

Cointegration equation	Cointegration equation 1		
Lnprivate (−1)	1		
Lnpublic (−1)	−0.81		
	(0.14)		
	[−5.81]		
Lnsale (−1)	0.12		
	(0.20)		
	[0.63]		
Intercept	−2.55		
Error correction	D(lnprivate)	D(lnpublic)	D(lnsale)
Cointegration equation 1	−0.10	0.02	−0.00
	(0.03)	(0.01)	(0.01)
	[−3.58]	[2.70]	[−0.49]
D(lnprivate(−1))	−0.20	−0.02	0.01
	(0.06)	(0.01)	(0.01)
	[−3.45]	[−1.72]	[0.87]
D(lnpublic(−1))	0.29	0.04	−0.01
	(0.27)	(0.06)	(0.06)
	[1.08]	[0.67]	[−0.16]
D(lnsale(−1))	0.28	0.07	0.15
	(0.26)	(0.06)	(0.06)
	[1.10]	[1.09]	[2.52]
Intercept	0.05	0.04	0.06
	(0.03)	(0.01)	(0.01)
	[1.71]	[6.22]	[7.72]
R^2^	0.12	0.04	0.03
Adj. R^2^	0.10	0.02	0.01
Sum sq. resids	27.52	1.57	1.56
SE equation	0.34	0.08	0.08
F-statistic	8.00	2.57	1.87
Log likelihood	−78.92	279.44	280.00
IC	0.67	−2.20	−2.20
Schwarz SC	0.74	−2.13	−2.13
Mean dependent	0.07	0.05	0.07
SD dependent	0.35	0.08	0.08
Determinant resid covariance (dof adj.)	4.48 × 10^–06^		
Determinant resid covariance	4.22 × 10^–06^		
Log likelihood	482.77		
Akaike information criterion	−3.72		
Schwarz criterion	−3.46		

Standard errors in () & t-statistics in []

Sample adjusted for a period between 1984 and 2008

There are 250 observations included after adjustments

D(lnprivate): first difference of log private sector expenditure; D(lnpublic): first difference of log public sector expenditure; D(lnsale): first difference of log sales; Lnprivate (−1): log private sector expenditure with one year lag; Lnpublic (−1): log public sector expenditure with one year lag; Lnsale (−1): log sales with one year lag; D(lnprivate(−1)): first difference of log private expenditure with one year lag; D(lnpublic(−1)): first difference of log public expenditure with one year lag; D(lnsale(−1)): first difference of sales with one year lag

Where y_it_ is a 3 *×* 1 vector, *i* refers to disease area, *t* refers to year, Γ is a matrix of autoregression coefficients, α is the vector of equilibrium-correction coefficients that adjust for short-run departures from the long-run (cointegration) equation, μ is the vector of intercepts in the cointegration equation, and γ is a vector of drift terms.

The results suggest that there is one cointegration relationship between the three variables. In the long run, public sector expenditure and private sector expenditure are complements and a 1 % increase in public sector expenditure is associated with an increase of 0.81 % in private sector expenditure. The long-run relationship between private sector expenditure and global pharmaceutical sales is not statistically significant.

The three coefficients on cointegration Eq. 1 suggest how each of the three variables responds to a deviation from long-run equilibrium. A positive deviation from equilibrium, which could be the result of a relative excess of private sector investment in R&D, leads to an increase of public sector expenditure and a decrease of private sector itself from the relative excess of private investment. However, global pharmaceutical sales do not respond to any deviation from equilibrium.

Table 4 reports results from adapting the best model to estimate the elasticity of private R&D expenditure with respect to government research expenditure and with respect to charity sector research expenditure separately. The results suggest that, in the long run, a 1 % increase in government sector expenditure is associated with a 0.66 % increase in private sector expenditure. Similarly, a 1 % increase in charity sector expenditure (which has been somewhat smaller than government expenditure) is associated with a 0.21 % increase in private sector expenditure. If the two components of public expenditure were in fixed proportions over time, the two estimated elasticities would sum to 0.81, which is approximately the case here. Global pharmaceutical sales have no significant relationship with the private sector expenditure in the long run.

**Table 4:** Modelling government expenditure and charity expenditure as two separate variables

Cointegration equation	Cointegration equation 1			
Lnprivate (−1)	1			
Lngoverment (−1)	−0.66			
	(0.18)			
	[−3.57]			
Lncharity (−1)	−0.21			
	(0.09)			
	[−2.36]			
Lnsale (−1)	0.16			
	(0.21)			
	[0.75]			
Intercept	−3.34			
Error Correction	D(Lnprivate)	D(Lngoverment)	D(Lncharity)	D(Lnsale)
Cointegration equation 1	−0.08	0.01	0.14	−0.00
	(0.03)	(0.01)	(0.05)	(0.01)
	[−2.99]	[1.63]	[3.08]	[−0.47]
D(lnprivate(−1))	−0.20	−0.01	−0.21	0.01
	(0.06)	(0.01)	(0.10)	(0.01)
	[−3.38]	[−0.74]	[−2.00]	[1.05]
D(lngoverment(−1))	0.10	−0.16	−0.04	−0.08
	(0.30)	(0.07)	(0.52)	(0.07)
	[0.34]	[−2.45]	[−0.07]	[−1.17]
D(lncharity(−1))	−0.01	−0.00	−0.11	0.01
	(0.04)	(0.01)	(0.06)	(0.01)
	[−0.34]	[−0.12]	[−1.82]	[1.60]
D(lnsale(−1))	0.30	0.02	0.18	0.16
	(0.26)	(0.06)	(0.44)	(0.06)
	[1.16]	[0.40]	[0.40]	[2.55]
Intercept	0.06	0.04	0.15	0.06
	(0.03)	(0.01)	(0.05)	(0.01)
	[2.10]	[5.91]	[3.05]	[7.98]
R^2^	0.10	0.04	0.06	0.05
Adj. R^2^	0.08	0.02	0.04	0.03
Sum sq. resids	27.97	1.30	81.67	1.53
SE equation	0.34	0.07	0.58	0.08
F-statistic	5.49	1.82	2.95	2.30
Log likelihood	−80.96	302.94	−214.88	281.99
Akaike information criterion	0.70	−2.38	1.77	−2.21
Schwarz SC	0.78	−2.29	1.85	−2.12
Mean dependent	0.07	0.03	0.14	0.07
SD dependent	0.35	0.07	0.59	0.08
Determinant resid covariance (dof adj.)		1.23 × 10^–06^		
Determinant resid covariance		1.12 × 10^–06^		
Log likelihood		294.17		
Akaike information criterion		−2.13		
Schwarz criterion		−1.73		

Standard errors in () & t-statistics in []

Sample adjusted for a period between 1984 and 2008

There are 250 observations included after adjustments

D(lngoverment): first difference of log government expenditure; D(lncharity): first difference of log charity expenditure; D(lnprivate): first difference of log private sector expenditure; D(lnsale): first difference of log sales; Lngoverment (-1): log government expenditure with one year lag; Lncharity (-1): log charity expenditure with one year lag; Lnprivate (−1): log private sector expenditure with one year lag Lnsale (−1): log sales with one year lag; D(lngoverment(−1)): first difference of log government expenditure with one year lag; D(lncharity(−1)): first difference of log charity expenditure with one year lag; D(lnprivate(−1)): first difference of log private sector expenditure with one year lag; D(lnsale(−1)): first difference of sales with one year lag

To test the sensitivity of our results to a possible shift in the relationship in 1993, we included two policy dummies as exogenous variables. The results are reported in Additional file 8. The key elasticity estimate is decreased slightly, from 0.81 to 0.68, as a result. The policy dummy that controls the trend effect of year 1993 is significant in the short-run equation for public expenditure but not global pharmaceutical sales nor private sector expenditure. The results suggest that the policy dummy in 1993 raises the change in log public expenditure by 0.04. The policy dummy that controls for the 1 year effect in 1993 is not statistically significant in the short-run equation for public sector expenditure.

#### Impulse response function

**Fig. 6:**
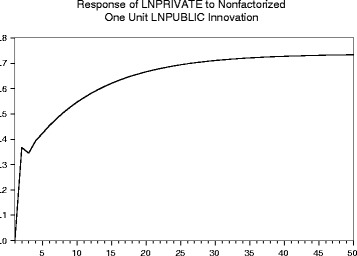
Impulse response of an increase in public research expenditure on private research and development expenditure

Figure 6 shows that more than half of the response from private sector expenditure as the result of a long-run shock to public sector expenditure will happen within the first 5 years (see Table F in Additional file 7 for the underlying data). It would take decades for private sector expenditure to move back to the equilibrium if there were no further shocks. One unit increase in the log public sector expenditure leads to 0.42 unit increase in the log private sector expenditure within the first 5 years. It will eventually lead to a 0.74-unit increase in the log private sector expenditure. This figure of 0.74 is lower than the long-run elasticity estimated to be 0.81, because the endogeneity of public expenditure in the model causes the 1 % shock to relax back to 0.91 % over time. We do not think this feature of the recent historical behaviour of these variables is relevant for policy, so the long-run elasticity of 0.81 (=0.74/0.91) is our best estimate.

## Discussion

In this section of the paper we discuss the limitations of the study, present possible policy interpretations of our empirical findings, and set out some topics for future research that we have identified as a result of the present study.

### Limitations of the study

Our study is inevitably limited by the extent and quality of the data that can be pulled together. Compilation of series of annual data since 1982 for government and charity expenditure, respectively, on biomedical and health research in the UK, by disease area, was a major task drawing on numerous sources. Inevitably, there is a risk that disease categorisations vary over time and between organisations. We aimed to mitigate this potential issue by relying wherever possible on the HRCS classification. Furthermore, some data points had to be extrapolated in order to have complete funding streams available over time. Nevertheless, the scope of data we gathered over the course of this project is unique and will be useful for further research.

Data for the disease area split of total pharmaceutical industry R&D expenditure in the UK do not exist. A major element of the study was to test whether it was possible to proxy the disease area split by observing the disease area split of later publications with pharmaceutical industry authors giving UK addresses. This proxy approach appears to have been successful. It allows for the trend increase over time in research publications by all types of authors, by focusing on the share of publications in each disease area each year rather than the absolute number.

We considered the possibility that different pharmaceutical companies, which concentrate their respective R&D programmes on different mixes of disease areas, may differ in their propensity to encourage their research staff to publish. If that were the case, the disease split of company-authored papers would over-represent the disease areas favoured by high-propensity-to-publish companies and under-represent the disease areas favoured by low-propensity-to-publish companies. In the period 1982–2012, there were of the order of 10 billion biomedical research papers in Web of Science, of which several hundred thousand can be expected to have one or more company authors – based on a preliminary assessment undertaken for us by CWTS at the University of Leiden. The cost and time involved in extracting and cleaning company names from these and linking those company names to company names in earlier years (allowing for mergers and name changes) would have been exorbitant, so we were unable to pursue this avenue. However, we have no reason to expect company propensity to publish to be correlated with company disease area preference for R&D, and hence no reason to expect bias in our results.

An alternative proxy for the disease split of private pharmaceutical industry R&D in the UK might be the disease split of patents citing authors with UK company addresses. We investigated this option but found that the proportion of patents identified to the nine named disease areas (Cardiology, Dermatology, Gastroenterology, Haematology, Infectious, Neurosciences, Oncology, Ophthalmology, Respiratory) was worryingly low in some years – below 10 % – and furthermore that there were implausibly large changes in this proportion from year to year. We therefore judged that the patent data would not be suitable as a way to proxy the disease area split of pharmaceutical industry R&D expenditure in the UK.

Our data set includes 10 disease areas. An obvious question concerns the heterogeneity of the data. We have experimented with various econometric approaches to account for this issue in our estimates. However, all approaches were undermined by the small number of observations at each disease level (n = 27 in the best-fit model). We therefore decided to estimate a model by pooling all data from the 10 diseases rather than adopting a model that addressed the heterogeneity of the data. This limitation of the model should be noted.

Furthermore, we pooled data from different disease areas and built a model that assumes that expenditures across the areas are determined independently but in a similar fashion. A pharmaceutical company’s revenues from sales in all of the disease areas where it currently has products are in principle available to fund any of its current R&D expenditure regardless of disease area. However, global sales in a particular disease area are an indication of the importance of that market at that time as a target for the pharmaceutical industry. Further, there are no better data available to proxy the supply of funding for disease-specific private R&D investment than disease-specific sales, and only a short historical record was available to construct a dynamic model, forcing us to tightly control the number of parameters to be estimated.

Finally, it should be noted that coefficients of the error correction terms, in Table 3, that embody the short-run adjustment towards the long-run equilibrium described by the cointegration equation suggest that both private sector and public sector R&D expenditures respond to any departure from the long-term equilibrium, but that sales do not. When there is a negative departure from the long-term equilibrium (that is, private sector R&D lies below its equilibrium level), private sector R&D expenditure responds strongly by increasing. In contrast, public sector R&D expenditure responds to such a departure by decreasing slightly. Notice that the response is five times stronger for the private sector than for public sector R&D expenditure.

### Comparison with previous work

Compared to the previous literature, i.e. Toole [5] and Ward and Dranove [6], the VECM has some advantages. First, the VECM provides estimates for both the long- and short-run relationships between variables. Ignoring cointegration relationship(s) in modelling time series data will, in general, lead to biased estimates. It is unclear from Toole’s analysis whether there is a long-run equilibrium relationship between variables [5]. Second, the econometric approaches adopted by Toole and by Ward and Dranove both assume that public research investment is exogenous to private sector investment; this strong assumption was self-criticised by Toole in the limitation section of his paper. An advantage of using the VECM is that it allows us to assume that private and public sector expenditure are both endogenous.

Toole adopted a polynomial lag structure in the model specification. The maximum number of lags chosen by Toole to be included, i.e. 8 years, is not fully explained by the author. The impact of this decision (the selection of the number of lags) on the predictions of Toole’s model is unclear but could be substantial given the quadratic lag function. In contrast, the VECM allowed the data to decide the number of lags to be included in a transparent way.

Ward and Dranove used a generalised least squares seemingly unrelated regression (SUR) to model government R&D expenditure, number of medical journal articles in MEDLINE and industry R&D expenditure. A clear advantage of this approach is that it allows for autocorrelation across equations and more importantly possible heteroskedasticity across disease categories. However, relative to the VECM approach we have adopted, there are three disadvantages of applying this approach in modelling the R&D expenditure time series data. First, all independent variables in the SUR models are assumed to be exogenous, which implicitly suggests that direct government R&D expenditure is an exogenous variable in modelling industry R&D expenditure. Our approach avoids the need to make that strong assumption. Second, in modelling the time series data, the generalised least squares SUR approach did not address the possible long-term relationship between variables, but short-term effects only. Third, the equation for industry R&D expenditure includes 7 years’ lags of US National Institutes of Health (NIH) research expenditures as independent variables. The authors self-criticised this specification that the “*lagged value of NIH’s expenditure exhibit a high degree of collinearity which yields large sampling variances and greater sensitivity of the individual coefficient estimates to model specification changes*” [6].

### Geographical scope

Our focus has been explicitly limited to the link between public and private research within the UK. We have not attempted to quantify the impact that UK public biomedical and health research expenditure might have on industry R&D in the rest of the world, nor the impact of public research spending in other countries on industry R&D within the UK. To what extent those international linkages exist, in either direction, represents a set of interesting further research questions, but they are beyond the scope of the present study. Moreover, data unavailability seems likely to render quantification of such international linkages impractical at present.

### Interpreting our findings

Subject to these limitations and caveats, we have found a long-term equilibrium relationship between public biomedical and health research spending in the UK and private sector pharmaceutical industry R&D expenditure in the UK. The Granger causality test was applied to the private R&D and public research expenditure series. The null hypothesis of the test is that one variable does not ‘Granger cause’ the other variable, i.e. adds no predictive information not already present in the series’ own history. Our results rejected the null hypothesis at the 5 % level in both directions, which indicates that there might be a dual causal relationship between private R&D and public research expenditure. Given this, a cautious interpretation of our subsequent model-based findings would be to say that we find a positive association between additional public research spend and additional private R&D.

We find that public research ‘crowds in’ private R&D rather than crowding it out. A 1 % increase/fall in public (meaning government plus charity) biomedical and health research spend in the UK eventually is associated, in our most robust model, with a 0.81 % increase/fall in pharmaceutical industry R&D in the UK. A sensitivity test using dummy variables for 1993 and from that year onwards produced a similar result with an elasticity of 0.68. We found a larger elasticity for pharmaceutical industry R&D with respect to additional government research expenditure than with respect to additional charity funded research, partly explained by the greater scale of government than charity research.

In 2012, the most recent year for our data, UK government plus charity research spend in the relevant fields was £3.43bn, with £1.22bn from charity sector and £2.21bn from government.^5^ In the same year, pharmaceutical industry R&D in the UK was £4.21bn. Given the relative magnitude of public and private research spending in the UK in our latest year of data (2012), the elasticity of 0.81 implies that a £1.00 increase in public biomedical and health research spend would result ultimately in a £0.99 increase in private pharmaceutical R&D in the UK (0.81 × 4.21/3.43 = 0.99). The 0.68 elasticity from the sensitivity analysis with dummy variables would correspondingly imply that on the same basis a £1.00 increase in public research spend would lead to a £0.83 increase in private R&D.

However, the elasticity of 0.81 has been calculated from expenditure data over the whole of the period 1982–2008, during which time the balance between public and private research spending has changed. Over the 27 years 1982–2008 as a whole, the ratio of aggregate private R&D to aggregate public research spend was a little lower than the ratio in 2012. A total of £73.2bn (in 2012 price terms) of private R&D over the period 1982–2008 and a total of £55.3bn of public research spend (government plus) charity, gives private R&D as 32 % higher over that whole period than public research spend. By comparison, in 2012, private R&D was 23 % higher than public research spend. Applying the private:public ratio from the whole period 1982–2008 (rather than the ratio in the most recent year of data, 2012) would imply that an extra £1 of public research would have stimulated an extra £1.07 (=0.81 × 73.2/55.3) of private R&D. If the lower 0.68 elasticity from the sensitivity test with dummy variables were used, the corresponding implication would on this basis be that a £1.00 increase in public research spend would lead to a £0.90 increase in private R&D.

Based on the most recent year’s data (2012), the elasticity of 0.66 from government research spending alone would imply that a £1.00 increase in government spending in biomedical and health research would result in a £1.26 increase in private pharmaceutical R&D in the UK (0.66 × 4.21/2.21 = 1.26). The elasticity of 0.21 from the charity sector would imply on the same basis of 2012 expenditure relativities that a £1.00 increase in charity spending in biomedical and health research would result in a £0.72 increase in private pharmaceutical R&D in the UK (0.21 × 4.21/1.22 = 0.72).

However, the relativities between charity, government and private research spend have changed over time. Applying the same elasticities to the aggregates of charity, government and private research spend, respectively, across the 1982–2008 period for which those elasticities have been estimated (rather than to the 2012 expenditures alone), would imply that an extra £1 of government research spend would stimulate £1.24 (=0.66 × 73.2/38.9) of extra private R&D, and that an extra £1 of charity research would stimulate £0.94 (=0.21 × 73.2/16.3) of extra private R&D.

The reasons for this modest difference in the scale of extra private R&D that is implied would be generated by an extra £1 of government research or charity research, respectively, would be an interesting focus for future research. Possible reasons may lie in different mixes or types of research being funded by government as compared with charity, e.g. a different basic/clinical mix – Toole [5] found that public basic research stimulated considerably more dollars of private R&D than did an equal amount of clinical research. It may also be that the government and charity sectors have, to differing degrees, explicit matched funding arrangements with the private sector. We hope to be able to investigate these issues further in future research.

The impulse response function from our most robust model implies that 44 % of the impact on private R&D would be seen in the first year following a change in public research spending. The remainder of the effect would take many years, even decades to fully work through.

Our UK-specific and up-to-date estimate of the elasticity of pharmaceutical industry R&D to a change in public research spending is different from the corresponding US elasticities found by Toole [5] and Ward and Dranove [6]. Ward and Dranove estimated that a 1 % change in government-funded medical research in the US would stimulate a 2.5 % change in US pharmaceutical industry R&D. Toole found an elasticity of 1.69 for the impact of US public basic research on pharmaceutical industry R&D in the US and 0.40 for clinical research. Assuming that at least half of public research is basic, Toole’s results imply an overall elasticity of 1.05 or more; this compares with our main finding of an elasticity of 0.81. A more modest elasticity in the UK than the US is consistent with the UK being a smaller and more open economy than the US, meaning that a smaller percentage of spillovers from public research would be expected to be captured within the UK and a higher percentage would leak out to industry in other countries.

The lower UK magnitude of elasticity has implications for the findings of the HERG et al. [4] estimate of the RoR to public biomedical and health research. That study estimated an economic RoR to UK public medical research, excluding the health gains to patients, of approximately 30 %, and cited a range from 26 % to 34 % depending on whether the elasticity assumed is, respectively, that found (for the US) by Toole [5] or that found by Ward and Dranove [6]. That calculation assumed a 50 % social RoR to private R&D spending, based on taking an average of the findings of a review of the empirical literature [4]. We have not revisited that social RoR figure, but for illustrative purposes if it were combined with the elasticity of 0.81 that we find, and with the relative scales of private and public research spending that existed in the UK in 2012, then the result would imply that the economic RoR (excluding health gains to patients) to public biomedical and health research in the UK would be 17 % (real, per annum). In other words a £1 investment in UK public biomedical and health research would be expected to benefit the UK economy as a whole to an extent equivalent to receiving 17 pence per year interest for ever in return for that £1 investment. Using the relative aggregate spends of the public and private sectors over the whole 1982–2008 period would result in a slightly higher RoR of 18 %.

The sensitivity analysis including dummies for 1993 and thereafter yielded a slightly lower elasticity of private R&D with respect to public research spending and would imply, on the same calculation basis, an economic RoR to public medical research equal to 15–16 % real per annum (where the lower number assumes 2012 relative public:private expenditure, and the higher number assumes 1982–2008 aggregate relativities between public and private expenditure).

Thus, a revised estimate of the real RoR to UK public medical research spending would appear from our results to lie in the range 15–18 %. Combined with our earlier estimates of the net monetary benefit of health gains arising cardiovascular research [4] and cancer [8], this would suggest a total RoR of between 24 % and 28 % arising from government and charitable investments in research.

### Future research

Using the dataset created for this study there is an additional key research question that we will be able to investigate in subsequent research. That is, what is the magnitude of the effect of charity biomedical and health research expenditure in the UK on government biomedical and health research expenditure and vice versa?

Additionally, further thought and investigation needs to be focused on how ‘spillover’ effects manifest themselves. That is, to gain a better understanding about how the additional private sector R&D in the UK comes about when UK public research spending increases. This is an idea that was previously raised in a workshop on spillovers (along with undertaking a UK biomedical and health specific study as reported here) [43]. This is likely to involve qualitative research into mechanisms that generate spillover effects and the barriers that hinder them. In particular, research is needed into how these effects are channelled through individuals and their formal and informal interactions, including, but not limited to, research collaborations and the labour market.

## Conclusion

The objective of this study was to estimate the magnitude of the effect of government and charity biomedical and health research expenditure in the UK, separately and in total, on subsequent private pharmaceutical sector R&D expenditure in the UK. By developing an econometric model to examine the statistical relationship between time series of biomedical and health research expenditures in the UK since 1982, and for different disease areas and sectors, we are able to infer a number of key findings relevant to UK biomedical and health research:

1. Public research investments ‘crowd in’ additional private sector R&D investments: every additional £1 of public research expenditure is associated with an additional £0.83–£1.07 of private sector R&D spend;
2. 44 % of that additional private sector expenditure occurs within 1 year, with the remainder accumulating over decades;
3. This spillover effect implies a real annual RoR (in terms of economic impact) to public biomedical and health research in the UK of 15–18 %.
4. When combined with previous estimates of the health gain that results from public medical research in cancer and CVD, the total RoR would be around 24–28 %.

Overall, this would suggest that historical returns from UK government and charity funded research in the UK compare favourably with the rates of return achieved on investments in the rest of the UK economy and are greatly in excess of the 3.5 % real annual RoR required by the UK government to public investments generally [44].

## Box 1: An overview of the modelling approach

- The econometric model is designed to capture salient features of the relationship that exists between various time series and is only as good as the data that are used and relies on the abstraction of the underlying phenomenon under study.
- Vector autoregression (VAR) is a commonly used approach for describing the evolution of multiple time series data.
- A vector error correction model (VECM) is a restricted form of VAR model that is appropriate when non-stationary time series data are found to have one or more cointegration relationship(s):
  - O A non-stationary time series is one where the mean and variance are not constant over time;
  - O If a combination of two or more non-stationary time series is stationary, then those series are said to have a cointegration relationship.
- A VECM provides estimates for the short-run dynamics and the long-run relationships between variables (cointegration).

## Additional files

Additional file 1:
**Proportion of publications with an author giving a UK industry address and proportion with an author giving a UK non-industry address, by disease area.** (XLSX 21 kb) Additional file 2:
**List of keywords used to identify relevant Wellcome Trust grants, by disease area.** (XLSX 41 kb) Additional file 3:
**Medical research charities allocated to one of nine Health Research Classification System (HRCS) codes.** (XLSX 11 kb) Additional file 4:
**Literature review of the drivers of private pharmaceutical R&D.** (DOCX 33 kb) Additional file 5:
**Interview protocol for key informants.** (DOCX 15 kb) Additional file 6:
**Estimated R&D expenditure for nine disease areas for government, charity and private sectors.** (XLSX 32 kb) Additional file 7:
**Modelling specification tables.** (DOCX 21 kb) Additional file 8:
**Model sensitivity analysis – including two policy dummies as exogenous variables.** (DOCX 18 kb)

## Abbreviations

ADF: Augmented Dicky Fuller
AMRC: Association of Medical Research Charities
ATC: Anatomical therapeutic classification
CE: Cointegration equation
CNS: Central nervous system
CVD: Cardiovascular disease
CWTS: Centre for Science and Technology Studies (Leiden University)
DH: Department of Health (England)
GDP: Gross domestic product
HEFCE: Higher Education Funding Council for England
HRCS: Health Research Classification System
MRC: Medical Research Council
NHS: National Health Service
NIH: National Institutes of Health
R&D: Research and development
RoR: Rate of return
SET: Science, engineering and technology
SUR: Seemingly unrelated regression
UK: United Kingdom
US: United States of America
VAR: Vector autoregression
VECM: Vector error correction model

## Footnotes

1 The return for mental health was estimated to be approximately 7 %. However, this was based on a more limited analysis due to data limitation and uncertainties around the effects of interventions in mental health and was presented with less confidence than the estimates for CVD.

2 For the finalised time series we used and presented calendar years. The first calendar year of a financial year was used. That is, financial year 1991/1992 is presented as calendar year 1991.

3 It should be noted that in the studies on cardiovascular research [4] and cancer [8] we used the inclusive measure as we wanted to err on the side of caution in calculating a rate of return, potentially overestimating investments.

4 CWTS is an interdisciplinary institute at Leiden University providing data products and services to a variety of research institutes. The institute utilises a bibliometric data system (based on an enhanced version of Thomson Reuters’ Web of Science database) enabling high-value bibliometric analyses. See http://www.cwts.nl/Home for more detail.

5 Note that there is no general consensus on the total UK government biomedical research spend. However, our bottom-up estimate for government expenditure is in the same ballpark with an estimate by the UK Clinical Research Collaboration (2012). Using 2009/2010 data, the UK Health Research Analysis 2009/2010 report estimates governmental spend (without charity) to be £1.304bn. Including spend on biomedical research by the Funding Councils in that year (£590m in 2009/2010) and, translating into 2012 constant prices, that figure becomes £2.032bn.
