# Supplementary material for: Quantifying the economic impact of government and charity funding of medical research on private research and development funding in the United Kingdom
Source: BMC Med. 2016 Feb 24;14:32. doi: 10.1186/s12916-016-0564-z (PMC4765095; doi:10.1186/s12916-016-0564-z)
Supplement: Additional file 4: — Literature review of the drivers of private pharmaceutical R&D. (DOCX 33 kb) [file 12916_2016_564_MOESM4_ESM.docx]

# Additional File 4: Literature review of the drivers of private pharmaceutical R&D

We (MP) conducted a literature review to identify drivers of private pharmaceutical R&D to ascertain whether it would be desirable to include further, control variables in the econometric model.

**Strategy:** We considered the first 30 hits in Google Scholar using the following keyword combinations:

- drivers; pharmaceutical; private research
- drivers; pharmaceutical; private R&D
- drivers; drug; private research
- drivers; drug; private R&D
- determinants; pharmaceutical; private research
- determinants; pharmaceutical; private R&D
- determinants; drug; private research
- determinants; drug; private R&D

To select the papers:

1. We first screened the Google Scholar hits according to the title
2. Then we selected the potentially relevant papers depending on the abstracts.

This strategy identified 17 papers. We added to this list two other papers (Cerda 2007; Dubois et al.2011) recommended by a member of the research team (JM-F). The 19 references are listed at the end of this Annex.

We reviewed the articles to identify the drivers, the measures and some possible instrumental variables as described by the authors. Three studies (Becker 2013; David et al. 2000; Guellec and Van Pottelsberghe De La Potterie 2003) were not specific to pharma R&D but describe the drivers of R&D in general. We nevertheless included them because they could provide useful information. One study in particular (David et al. 2000) reviews econometric studies regressing private R&D on public R&D^[[1]](#footnote-1)^. Another study (Henderson and Cockburn 1996) does not describe the drivers of pharmaceutical R&D but the drivers of pharmaceutical R&D *productivity*.

**Findings:** The tables below report the findings from the literature review, organising the different drivers into five categories:

- market structure
- regulatory regime
- location and resources related variables
- foreign spillovers
- public investment

# Market structure

| Drivers | Variables | Definitions | Data type | References |
| --- | --- | --- | --- | --- |
| global market size | potential market size driven by demographic and income changes | the potential market size is defined by:  $M_{it}=\sum_{a} u_{ia} {inc}_{at}$  where ${inc}_{at}$ is the income of individuals in age group $a=\left\{ 0-4, \ldots, 85+ \right\}$ at time *t*  $u_{ia}$ is the average expenditure share of drugs in indication *i* in the total income of those in age group *a* | continuous | [Acemoglu and Linn 2004] |
|  | potential market size per medical indication driven by demographic and income changes weighted by prevalence rate in a given disease | the potential market size per medical indication *i* in year *t* is defined by:  ${MKS}_{it}=\sum_{a} w_{iat} {pop}_{at} {inc}_{at}$  where ${pop}_{at}$ is the total population in age group $a=\left\{ 0-4, \ldots, 85+ \right\}$  ${inc}_{at}$ is the (median) individual income of persons in age group *a* in year *t*  $w_{iat}$ , a weight for prevalence rate of age-group *a* on disease *i* at time *t*, is computed as the number of individuals dying per age-group at time *t* of a specific disease *i* divided by the total number of deaths from disease type *i* at *t* | continuous | [Cerda 2007] |
|  | potential market size driven by demographic and income changes weighted for medical indication | the potential market size per medical indication *i* in year *t* is defined by:  ${MKS}_{it}=\sum_{a} w_{ia} {pop}_{at} {inc}_{at}$  where ${pop}_{at}$ is the total population in age group $a=\left\{ 0-4, \ldots, 85+ \right\}$  ${inc}_{at}$ is the (median) individual income of persons in age group *a* in year *t*  $w_{ia}$ , a weight for medical indication *i* in age group *a*, is computed as the average share of expenditure for drugs assigned to indication *i* divided by the total individual income of individuals in age group *a* | continuous | [Rake 2012] |
| unmet medical need | global disease burden as computed by the WHO | mortality and disease burden to society, as measured by days of life to either premature death or disability, weighted by severity | continuous | [Agarwal and Searls 2009] |
| commercial potential | developed world disease burden as computed by the WHO | mortality and disease burden to society in developed world, as measured by days of life to either premature death or disability, weighted by severity | continuous |  |
| availability of funds for R&D investment | gross sales revenue | total industry sales (per therapeutic class) in the UK. The nominal flows need to be deflated and referred to a base year | continuous | [Toole 2007]  [Dubois 2011] |

# Regulatory regime

| Drivers | Variables | Definitions | Data type | References |
| --- | --- | --- | --- | --- |
| property-rights regime | Heritage Foundation’s Freedom Index ranking countries according to their regulation, government intervention, and level of property-rights protection | The property rights component is an assessment of the ability of individuals to accumulate private property, secured by clear laws that are fully enforced by the state. It measures the degree to which a country’s laws protect private property rights and the degree to which its government enforces those laws. It also assesses the likelihood that private property will be expropriated and analyses the independence of the judiciary, the existence of corruption within the judiciary, and the ability of individuals and businesses to enforce contracts.  The more certain the legal protection of property, the higher a country’s score; similarly, the greater the chances of government expropriation of property, the lower a country’s score. Countries that fall between two categories may receive an intermediate score.  <http://www.heritage.org/index/property-rights> | categorical | [Civan and Maloney 2006] |
| regulatory policies | dummy for 1984 (Hatch-Waxman Act extending patent life) | dummy variable representing the years for which the Waxman-Hatch Act (enacted in 1984) was in effect | binary | [Giaccotto et al. 2005] |
| government R&D policies | tax credit/subsidy | dummy variable representing the years for which the PPRS R&D allowances might have an impact (need to test/decide between 1993, 1999, 2005).  1993: individually agreed targets but industry-wide allowance of 20% of sales.  1999: Up to 20% of sales (17% for assessing price increases). An additional 0.25% of sales for each ‘in patent molecule’ with in excess of £0.5m. Maximum of 3%. Overall maximum of 23%.  2005: Up to 20% of sales (15% for assessing price increases) 0.25% per ‘in patent active substance’ or equivalent with sales greater than £0.3m. Maximum of additional 5%. Different breakdown for companies in first three years within scheme (higher allowance per product but with same overall cap). Additional 1% per paediatric product, in the first year available on prescription, up to maximum of 3%. Not included when assessing price increases. Overall maximum of 28%. | binary | [Becker 2013] – this study is not specific to the pharma industry |

# Location and resource related variables

| Drivers | Variables | Definitions | Data type | References |
| --- | --- | --- | --- | --- |
| technological opportunities | rate of growth of knowledge stock | the knowledge stock ($K_{it}$) consists of scientific publications (${Pub}_{it}$) related to medical indication *i* and published in year *t* that are assigned to categories related to pharmaceutical research  knowledge stocks are created using the perpetual inventory method:  $K_{it}={Pub}_{it}+(1-\delta)K_{i,t-1}$  where $\delta$ is the depreciation rate of the knowledge stock (Cockburn and Henderson, 2001, set it to 20%).  the rate of growth of the technological stock (${TO}_{it}$) can be expressed as:  ${TO}_{it}=\frac{K_{it}-K_{i,t-1}}{K_{i,t-1}}*100$  the pre-sample rate of growth of the technological stock is constructed as the average annual growth rate of the knowledge stock in the 5 years pre-dating the example:  ${TO}_{i0}=\left( \left( \frac{K_{i,-1}}{K_{i,-5}} \right)^{\frac{1}{5}}-1 \right)*100$ | continuous | [Rake 2012] |
|  | pool of scientists | number of doctorates awarded each year by UK universities in biology and chemistry | discrete | [Agarwal and Searls 2009], [Becker 2013] – this study is not specific to pharma industry |
|  | global scientific activity | counts of articles returned by PubMed when restricted to individual years by the standard filter function for publication date | discrete | [Agarwal and Searls 2009] |

# Public investment

| Drivers | Variables | Definitions | Data type | References |
| --- | --- | --- | --- | --- |
| public basic research | basic research flow and stock by  therapeutic class | grants and contracts awarded by the Department of Health | continuous | [Toole 2007] |
| public clinical research | clinical research flow and stock by  therapeutic class | grants and contracts awarded by the Department of Health | continuous |  |

## References

Acemoglu, D. and Linn, J. 2004. Market Size in Innovation: Theory and Evidence from the Pharmaceutical Industry. *The Quarterly Journal of Economics* *119*, 3, 1049–1090.

Agarwal, P. and Searls, D.B. 2009. Can literature analysis identify innovation drivers in drug discovery? *Nature Reviews Drug Discovery* *8*, 11, 865–878.

Becker, B. 2013. The determinants of R&D investment: a survey of the empirical research. .

Blume-Kohout, M.E. and Sood, N. 2008. *The Impact of Medicare Part D on Pharmaceutical R&D*. National Bureau of Economic Research.

Book, R.A. 2002. Public Research Funding and Private Innovation: The Case of the Pharmaceutical Industry. http://rbook.freeshell.org/Book_PharmInnov_Short.pdf.

Cerda, R.A. 2007. Endogenous innovations in the pharmaceutical industry. *Journal of Evolutionary Economics* *17*, 4, 473–515.

Civan, A. and Maloney, M.T. 2006. The determinants of pharmaceutical research and development investments. *Contributions in Economic Analysis & Policy* *5*, 1.

David, P.A., Hall, B.H., and Toole, A.A. 2000. Is public R&D a complement or substitute for private R&D? A review of the econometric evidence. *Research Policy* *29*, 4, 497–529.

Dubois, P., De Mouzon, O., Scott-Morton, F., Seabright, P. 2011. Market size and pharmaceutical innovation. Institut d’Economie Industrielle Working Paper. Institut d’Economie Industrielle, University of Toulouse: Toulouse.

Giaccotto, C., Santerre, R.E., and Vernon, J.A. 2005. Drug Prices and Research and Development Investment Behavior in the Pharmaceutical Industry. *The Journal of Law and Economics* *48*, 1, 195–214.

Grabowski, H.G. 1968. The Determinants of Industrial Research and Development: A Study of the Chemical, Drug, and Petroleum Industries. *Journal of Political Economy* *76*.

Grabowski, H.G. and Vernon, J. 2001. The determinants of pharmaceutical research and development expenditures. In: P.D.D.C. Mueller and P.D.U. Cantner, eds., *Capitalism and Democracy in the 21st Century*. Physica-Verlag HD, 207–221.

Guellec, D. and Van Pottelsberghe De La Potterie, B. 2003. The impact of public R&D expenditure on business R&D. *Economics of Innovation and New Technology* *12*, 3, 225–243.

Henderson, R. and Cockburn, I. 1996. Scale, scope, and spillovers: the determinants of research productivity in drug discovery. *The Rand journal of economics*, 32–59.

Mahlich, J.C. and Roediger-Schluga, T. 2006. The Determinants of Pharmaceutical R&D Expenditures: Evidence from Japan. *Review of Industrial Organization* *28*, 2, 145–164.

Rake, B. 2012. *Determinants of pharmaceutical innovation: The role of technological opportunities revisited*. Jena Economic Research Papers.

Scherer, F.M. 2001. The Link Between Gross Profitability And Pharmaceutical R&D Spending. *Health Affairs* *20*, 5, 216–220.

Toole, A.A. 2007. Does Public Scientific Research Complement Private Investment in Research and Development in the Pharmaceutical Industry? *The Journal of Law and Economics* *50*, 1, 81–104.

Vernon, J.A. 2005. Examining the link between price regulation and pharmaceutical R&D investment. *Health Economics* *14*, 1, 1–16.

1. Control variables include:

   - size (firm or industry dummies)
   - sales
   - sales to government
   - cash flow
   - time dummies
   - geography dummies
   - patents
   - interest rate
   - HHI
   - country dummies

   [↑](#footnote-ref-1)
