# Supplementary material for: Quantifying the economic impact of government and charity funding of medical research on private research and development funding in the United Kingdom
Source: BMC Med. 2016 Feb 24;14:32. doi: 10.1186/s12916-016-0564-z (PMC4765095; doi:10.1186/s12916-016-0564-z)
Supplement: Additional file 5: — Interview protocol for key informants. (DOCX 15 kb) [file 12916_2016_564_MOESM5_ESM.docx]

# Additional File 5: Interview Protocol for key informants

## Introduction

Explain background to the project: public/charity/private spillovers are a big determinant of the return to medical research; providing up-to-date, and UK, quantification; have public/charity medical research spend by therapy area but not private industry R&D by therapy area; therefore having to find proxies for the therapy area split over time.

Ask: are you aware of any data for your company, or for the pharmaceutical industry more widely, that shows the split of company/industry **UK** R&D expenditure by therapy area over time. If so, could that data be provided to us?

Then, share our data on trends in public, charity and private medical research spending in the UK, and in the therapy area splits of papers by (UK-based) authors from the company, and ask the following questions.

## Validating the proxies

Thinking about your knowledge of your own company’s, and of any other pharmaceutical companies’, R&D expenditures ***in the UK***:

- Do publication or patent based proxies for the therapy area split look sensible for recent years (and as far back as you have knowledge)?
- Are you aware of any other information, about your company or the industry, that would help to identify the allocation of UK pharmaceutical industry R&D spending between therapy areas?

## Public+charity to private spillovers

To what extent would you expect a change in the therapy area focus or total level of public and charity funded medical research in the UK to produce a change in the therapy area focus of level of pharmaceutical industry R&D in the UK?

If at all, then why and over what time period would this effect play out?

What factors might affect the extent of such an effect? For example, are any specific therapy areas different from the others in this respect?

Would there be an impact on pharmaceutical industry R&D globally – either on the therapy area focus or the total level of pharmaceutical industry R&D globally?

If at all, then why and over what time period would this effect play out?

What factors might affect the extent of such an effect?

Are you aware of there having been (as far back as you can recall) any ‘shocks’ causing significant changes to the therapy area focus or scale of pharmaceutical industry R&D globally or in the UK specifically? For example: major policy shifts, major discoveries or scientific advances, changes of industry structure.

## Private to public+charity spillovers

To what extent would you expect a change in the therapy area focus or total level of pharmaceutical industry R&D in the UK to feed back to the therapy area focus or total level of public and charity funded medical research in the UK?

If at all, then why and over what time period would this effect play out?

What factors might affect the extent of such feedback?

Would you expect a change in the therapy area focus or total level of pharmaceutical industry R&D globally to feed back to the therapy area focus or total level of public and charity funded medical research in the UK?

If at all, then why and over what time period would this effect play out?

What factors might affect the extent of such feedback?

Are you aware of there having been (as far back as you can recall) any ‘shocks’ that you expect would have caused significant changes to the therapy area focus or scale of public+charity medical research expenditure in the UK? For example: major policy shifts, major discoveries or scientific advances.

## Next steps

Thanks for taking the time to be interviewed. We will write a note of key points from this interview and send you the draft for confirmation or correction, as appropriate.

We will shortly be conducting the econometric analysis. Outputs are planned to include a seminar/workshop in London when we have the econometric results: would you like to participate in that?
