# Supplementary material for: Quantifying the economic impact of government and charity funding of medical research on private research and development funding in the United Kingdom
Source: BMC Med. 2016 Feb 24;14:32. doi: 10.1186/s12916-016-0564-z (PMC4765095; doi:10.1186/s12916-016-0564-z)
Supplement: Additional file 7: — Modelling specification tables. (DOCX 21 kb) [file 12916_2016_564_MOESM7_ESM.docx]

# Additional File 7 – Modelling specification tables

## Table A: ADF unit root test

| Variables | ADF Fisher Chi-square | Probability |
| --- | --- | --- |
| In levels | | |
| Public | 0.62 | 1.00 |
| Government | 9.25 | 0.98 |
| Charity | 6.63 | 1.00 |
| Private | 21.54 | 0.37 |
| Sale | 0.87 | 1.00 |
| In first difference | | |
| D(Public) | 152.64 | 0.00 |
| D(Government) | 191.98 | 0.00 |
| D(Charity) | 115.80 | 0.00 |
| D(Private) | 186.05 | 0.00 |
| D(Sale) | 69.42 | 0.00 |
| In logs | | |
| Log(Public) | 3.04 | 1.00 |
| Log(Government) | 11.96 | 0.92 |
| Log(Charity) | 9.63 | 0.97 |
| Log(Private) | 35.07 | 0.02 |
| Log(Sale) | 13.27 | 0.87 |
| In first difference of log | | |
| D(LogPublic) | 172.81 | 0.00 |
| D(LogGoverment) | 235.38 | 0.00 |
| D(LogCharity) | 146.94 | 0.00 |
| D(LogPrivate) | 215.36 | 0.00 |
| D(LogSale) | 140.56 | 0.00 |

## Table B: one lag in the VECM model

|  | Deterministic trend 1 | Deterministic trend 2 | Deterministic trend 3 | Deterministic trend 4 |
| --- | --- | --- | --- | --- |
| Co-integration rank | 2 | 2 | 1 | 1 |
| Number of insignificant coefficients | 11 out of 15 | 12 out of 15 | 8 out of 15 | 8 out of 15 |
| Log likelihood | 453.83 | 459.32 | 457.35 | 457.40 |
| AIC | -2.99 | -3.01 | -3.03 | -3.02 |
| Schwarz Criterion | -2.72 | -2.72 | -2.80 | -2.78 |
| Residual autocorrelation test passed up to lag | 6 | 6 | 6 | 6 |

## Table C: two lags in the VECM model

|  | Deterministic trend 1 | Deterministic trend 2 | Deterministic trend 3 | Deterministic trend 4 |
| --- | --- | --- | --- | --- |
| Co-integration rank | 1 | 1 | 1 | 1 |
| Number of insignificant coefficients | 15 out of 21 | 16 out of 21 | 17 out 24 | 17 out of 24 |
| Log likelihood | 440.05 | 446.91 | 452.88 | 452.90 |
| AIC | -2.97 | -3.01 | -3.04 | -3.03 |
| Schwarz Criterion | -2.66 | -2.69 | -2.69 | -2.67 |
| Residual autocorrelation tests passed up to lag | 6 | 6 | 6 | 6 |

## Table D: three lags in the VECM model

|  | Deterministic trend 1 | Deterministic trend 2 | Deterministic trend 3 | Deterministic trend 4 |
| --- | --- | --- | --- | --- |
| Co-integration rank | 2 | NA | NA | 2 |
| Number of insignificant coefficients | 28 out of 33 |  |  | 27 out of 36 |
| Log likelihood | 440.80 |  |  | 453.97 |
| AIC | -2.98 |  |  | -3.04 |
| Schwarz Criterion | -2.46 |  |  | -2.45 |
| Residual autocorrelation tests passed up to lag | 6 |  |  | 6 |

## Table E: Specifications with assumed time lags between funding and publication from 0 to 5

| Time lag | Coefficient of the lnpublic (t) | Complement or substitution | Cointegration rank | Number of insignificant coefficients | Log likelihood | AIC | Schwarz Criterion | Residual autocorrelation tests passed up to lag |
| --- | --- | --- | --- | --- | --- | --- | --- | --- |
| t+0 | -0.38  (-3.22) | Complement | 1 | 8 out of 15 | 457.35 | -3.03 | -2.80 | 6 |
| t+1 | -0.88  (-6.20) | Complement | 1 | 9 out of 15 | 486.47 | -3.35 | -3.11 | 4 |
| t+2 | -1.12  (-5.89) | Complement | 1 | 7 out of 15 | 488.29 | -3.48 | -3.24 | 4 |
| t+3 | -0.87  (-6.14) | Complement | 1 | 8 out of 15 | 475.32 | -3.52 | -3.27 | 4 |
| t+4 | -0.81  (-5.81) | Complement | 1 | 9 out of 15 | 482.77 | -3.72 | -3.46 | 6 |
| t+5 | -0.74  (-5.41) | Complement | 1 | 9 out of 15 | 446.88 | -3.57 | -3.31 | 1 |

Table F: Response of private R&D expenditure to public R&D expenditure, private R&D expenditure and sale innovation

| Period | Response of LNPRIVATE to nonfactorized one unit LNPUBLIC innovation | Response of LNPRIVATE to nonfactorized one unit LNRIVATE innovation | Response of LNPRIVATE to nonfactorized one unit LNSALE innovation |
| --- | --- | --- | --- |
| 1 | 0.0000 | 1.0000 | 0.0000 |
| 2 | 0.3674 | 0.6978 | 0.2714 |
| 3 | 0.3451 | 0.6901 | 0.2441 |
| 4 | 0.3934 | 0.6289 | 0.2284 |
| 5 | 0.4236 | 0.5839 | 0.2049 |
| 6 | 0.4537 | 0.5411 | 0.1842 |
| 7 | 0.4803 | 0.5027 | 0.1651 |
| 8 | 0.5046 | 0.4678 | 0.1478 |
| 9 | 0.5266 | 0.4363 | 0.1322 |
| 10 | 0.5465 | 0.4077 | 0.1180 |
| 11 | 0.5645 | 0.3818 | 0.1052 |
| 12 | 0.5808 | 0.3584 | 0.0936 |
| 13 | 0.5956 | 0.3372 | 0.0831 |
| 14 | 0.6089 | 0.3180 | 0.0736 |
| 15 | 0.6211 | 0.3005 | 0.0649 |
| 16 | 0.6320 | 0.2848 | 0.0571 |
| 17 | 0.6420 | 0.2705 | 0.0500 |
| 18 | 0.6510 | 0.2576 | 0.0436 |
| 19 | 0.6591 | 0.2459 | 0.0378 |
| 20 | 0.6665 | 0.2352 | 0.0326 |
| 21 | 0.6732 | 0.2256 | 0.0278 |
| 22 | 0.6793 | 0.2169 | 0.0235 |
| 23 | 0.6847 | 0.2091 | 0.0196 |
| 24 | 0.6897 | 0.2019 | 0.0161 |
| 25 | 0.6942 | 0.1955 | 0.0129 |
| 26 | 0.6983 | 0.1896 | 0.0100 |
| 27 | 0.7020 | 0.1843 | 0.0073 |
| 28 | 0.7053 | 0.1795 | 0.0049 |
| 29 | 0.7083 | 0.1752 | 0.0028 |
| 30 | 0.7111 | 0.1712 | 0.0008 |
| 31 | 0.7136 | 0.1677 | -0.0009 |
| 32 | 0.7158 | 0.1644 | -0.0025 |
| 33 | 0.7178 | 0.1615 | -0.0040 |
| 34 | 0.7197 | 0.1588 | -0.0053 |
| 35 | 0.7214 | 0.1564 | -0.0065 |
| 36 | 0.7229 | 0.1543 | -0.0076 |
| 37 | 0.7242 | 0.1523 | -0.0085 |
| 38 | 0.7255 | 0.1505 | -0.0094 |
| 39 | 0.7266 | 0.1489 | -0.0102 |
| 40 | 0.7276 | 0.1475 | -0.0109 |
| 41 | 0.7285 | 0.1461 | -0.0116 |
| 42 | 0.7294 | 0.1449 | -0.0122 |
| 43 | 0.7301 | 0.1438 | -0.0127 |
| 44 | 0.7308 | 0.1429 | -0.0132 |
| 45 | 0.7314 | 0.1420 | -0.0137 |
| 46 | 0.7320 | 0.1412 | -0.0141 |
| 47 | 0.7325 | 0.1404 | -0.0144 |
| 48 | 0.7330 | 0.1398 | -0.0147 |
| 49 | 0.7334 | 0.1392 | -0.0150 |
| 50 | 0.7338 | 0.1386 | -0.0153 |
