# Supplementary material for: Quantifying the economic impact of government and charity funding of medical research on private research and development funding in the United Kingdom
Source: BMC Med. 2016 Feb 24;14:32. doi: 10.1186/s12916-016-0564-z (PMC4765095; doi:10.1186/s12916-016-0564-z)
Supplement: Additional file 8: — Model sensitivity analysis – including two policy dummies as exogenous variables. (DOCX 18 kb) [file 12916_2016_564_MOESM8_ESM.docx]

# Additional File 8: Sensitivity analysis – including two policy dummies as exogenous variables

| Cointegration equation | Cointegration equation 1 |  |  |
| --- | --- | --- | --- |
| Lnprivate (-1) | 1 |  |  |
| Lnpublic (-1) | -0.68  (0.13)  [-5.08] |  |  |
| Lnsale (-1) | -0.16  (0.21)  [-0.78] |  |  |
| Intercept | -0.38 |  |  |
| Error correction | D(lnprivate) | D(lnpublic) | D(lnsale) |
| Cointegration equation 1 | -0.13 | 0.01 | 0.00 |
|  | (0.03) | (0.01) | (0.01) |
|  | [-3.78] | [ 1.23] | [ 0.40] |
| D(lnprivate(-1)) | -0.19 | -0.02 | 0.01 |
|  | (0.06) | (0.01) | (0.01) |
|  | [-3.16] | [-1.53] | [ 0.54] |
| D(lnpublic(-1)) | 0.21 | -0.01 | -0.04 |
|  | (0.28) | (0.07) | (0.06) |
|  | [ 0.75] | [-0.21] | [-0.69] |
| D(lnsale(-1)) | 0.31 | 0.05 | 0.15 |
|  | (0.26) | (0.06) | (0.06) |
|  | [ 1.20] | [ 0.88] | [ 2.55] |
| Intercept | 0.03 | 0.02 | 0.05 |
|  | (0.04) | (0.01) | (0.01) |
|  | [ 0.73] | [ 2.45] | [ 5.24] |
| Policy dummy 1 | 0.03 | 0.04 | 0.01 |
|  | (0.05) | (0.01) | (0.01) |
|  | [ 0.59] | [ 3.33] | [ 0.67] |
| Policy dummy 2 | 0.11 | 0.00 | 0.14 |
|  | (0.11) | (0.03) | (0.02) |
|  | [ 1.04] | [ 0.10] | [ 5.90] |
| R-squared | 0.13 | 0.08 | 0.16 |
| Adj. R-squared | 0.11 | 0.05 | 0.14 |
| Sum sq. resids | 27.15 | 1.51 | 1.35 |
| S.E. equation | 0.33 | 0.08 | 0.07 |
| F-statistic | 5.91 | 3.33 | 7.66 |
| Log likelihood | -77.24 | 284.18 | 297.89 |
| AIC | 0.67 | -2.22 | -2.33 |
| Schwarz SC | 0.77 | -2.12 | -2.23 |
| Mean dependent | 0.07 | 0.05 | 0.07 |
| S.D. dependent | 0.35 | 0.08 | 0.08 |
| Determinant resid covariance (dof adj.) | | 3.80E-06 |  |
| Determinant resid covariance | | 3.49E-06 |  |
| Log likelihood |  | 506.51 |  |
| AIC |  | -3.86 |  |
| Schwarz criterion |  | -3.52 |  |

1. Standard errors in ( ) & t-statistics in [ ]
2. Sample adjusted for a period between 1984 and 2008.
3. There are 250 observations included after adjustments.
